# Decoupling Optical Functions via Ratio-Tunable Conjugated Copolymers with Hydrogen-Bond-Enforced Rigidity for Quenching-Resistant NIR-II Phototheranostics

**DOI:** 10.1021/acsnano.6c05195

**Published:** 2026-07-13

**Authors:** Weilong Chen, Chuang Zhang, Guan-Lin Wu, Ka-Wai Lee, Zhiqiang Guan, Yujuan Li, Bo-De Chen, Chao Zhao, Yung-Kang Peng, Jinfeng Zhang, Chu-Chen Chueh, Yingpeng Wan

**Affiliations:** † Center of Super-Diamond and Advanced Films (COSDAF), 53025 City University of Hong Kong, 83 Tat Chee Avenue, Kowloon, Hong Kong SAR 999077, P. R. China; ‡ Department of Chemistry, City University of Hong Kong, 83 Tat Chee Avenue, Kowloon, Hong Kong SAR 999077, P. R. China; § Key Laboratory of Molecular Medicine and Biotherapy School of Life Science, 47833 Beijing Institute of Technology Beijing 100081, P. R. China; ∥ Department of Chemical Engineering, 33561 National Taiwan University, Taipei 10617, Taiwan; ⊥ School of Life Science, Beijing Institute of Technology, Haidian, Beijing 100081, China

**Keywords:** NIR-II fluorescence imaging, phototherapy, conjugated copolymers, rigid segments, quenching-resistant

## Abstract

Organic near-infrared II (NIR-II) phototheranostic agents hold great promise for tumor imaging and phototherapy. However, their development is fundamentally challenged by the coupled and competing optical pathways in conventional designs: rigid planar fluorophores that excel in the molecular state often suffer from severe aggregation-caused quenching (ACQ) in nanoparticles, leading to drastic losses in both fluorescence and reactive oxygen species (ROS) generation. To decouple these optical functions, we report a modular copolymer-based anti-quenching strategy by integrating a flexible segment with a hydrogen-bond-enforced rigid segment. The key to decoupling lies in the tunable segment ratio, which allows composition-dependent modulation with reduced trade-offs of NIR-II fluorescence, ROS production, and photothermal conversion. As the rigid-segment fraction increases in the copolymer series (F8R2, F5R5, and F2R8), NIR-II fluorescence and ROS generation are markedly enhanced, while the photothermal conversion efficiency is only slightly compromised. Remarkably, the F2R8 nanoparticles (80% rigid segment) retain >60% of their NIR-II fluorescence and high ROS productivity in the aggregated state, starkly contrasting the >90% quenching observed in conventional ACQ-type small molecules. This effective partial decoupling of optical functions and suppression of ACQ enable high-contrast NIR-II fluorescence imaging-guided combined phototherapy *in vivo*. Our work provides a general design paradigm based on copolymer modularity for developing high-performance quenching-resistant organic phototheranostics.

## Graphical abstract

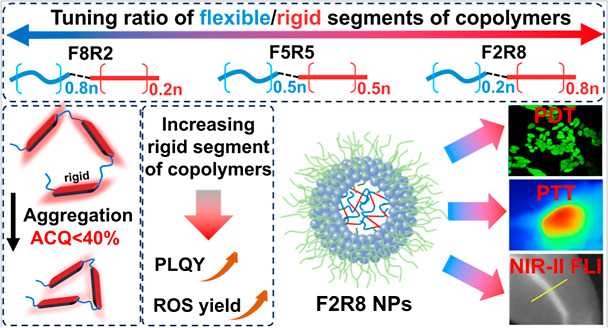

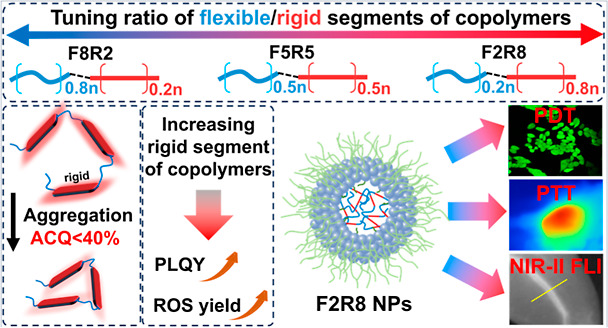

## 1 Introduction

Near-infrared-II (NIR-II, 1000–1700 nm) fluorescent phototheranostic agents have emerged as powerful tools in precision oncotherapy, enabling deep-tissue optical imaging with minimized scattering and autofluorescence, together with noninvasive photodynamic (PDT) and photothermal (PTT) treatments.^1–7^ These capabilities have driven intense interest in developing organic NIR-II materials that simultaneously offer strong NIR absorption, high fluorescence quantum yields (QYs), and efficient generation of therapeutic outputs such as reactive oxygen species (ROS) or heat.^8–12^ However, achieving such a balanced photophysical performance in bioavailable nanoparticle states remains a challenge. Many organic chromophores exhibit excellent fluorescence or ROS generation in dilute molecular solutions but lose these desirable properties upon nanoformulation, severely limiting their performance *in vivo*.^13–16^

A widely adopted molecular design principle for enhancing NIR absorption, intersystem crossing (ISC), and fluorescence emission is to enforce rigid, planar π-conjugated structures, as exemplified by commercial photosensitizers and dyes such as zinc phthalocyanine (ZnPc) and heptamethine/aza-BODIPY-derived analogues.^17,18^ In the molecularly dissolved state, rigid planarization can promote strong oscillator strength and facilitate efficient ROS generation or long-wavelength emission.^19,20^ However, these same structural features often become detrimental in aqueous environments: upon nanoparticle formation, such chromophores tend to pack tightly through intermolecular π–π stacking, leading to pronounced aggregation-caused quenching (ACQ).^21^ This behavior mainly arises because strong intermolecular interactions induce excitonic coupling and the formation of nonemissive aggregates, while dense packing promotes nonradiative energy dissipation pathways that suppress radiative decay. In addition, restricted oxygen diffusion and limited accessibility of excited triplet states in tightly packed structures can hinder ROS generation. As a result, both fluorescence QY and ROS generation efficiency can decrease dramatically by over 90% in the aggregated state, compromising imaging contrast and therapeutic potency (Figures 1 and S1–S5). Although diverse molecular and nanoengineering strategies, including the introduction of steric hindrance, modulation of coplanarity, and the formation of protein complexes, can alleviate ACQ to some extent, their resistance to quenching remains limited, underscoring the need for more effective design principles.^9,22^ Aggregation-induced emission (AIE) materials have greatly advanced anti-ACQ molecular design and have produced many high-performance luminogens across the visible and NIR spectral regions.^23–25^ Their success illustrates the value of restricting intramolecular motion to maintain radiative decay in aggregated states.^26^ Rather than replacing this well-established paradigm, complementary strategies that preserve the intrinsic advantages of rigid chromophores while mitigating aggregation-induced losses are also of considerable interest. In particular, rigid planar chromophores often possess strong absorption coefficients and extended conjugation beneficial for long-wavelength excitation and photothermal conversion, yet remain susceptible to ACQ in nanoparticle states. Therefore, developing alternative approaches that can retain these favorable characteristics while improving aggregation tolerance is highly desirable. In NIR-II phototheranostics, achieving simultaneous optimization of fluorescence brightness, ROS production, and photothermal efficiency often requires complementary design strategies beyond small-molecule luminogens. Within this context, polymeric systems provide unique opportunities to decouple and adjust multiple optical processes within a single platform.^27–31^

**Figure 1:**
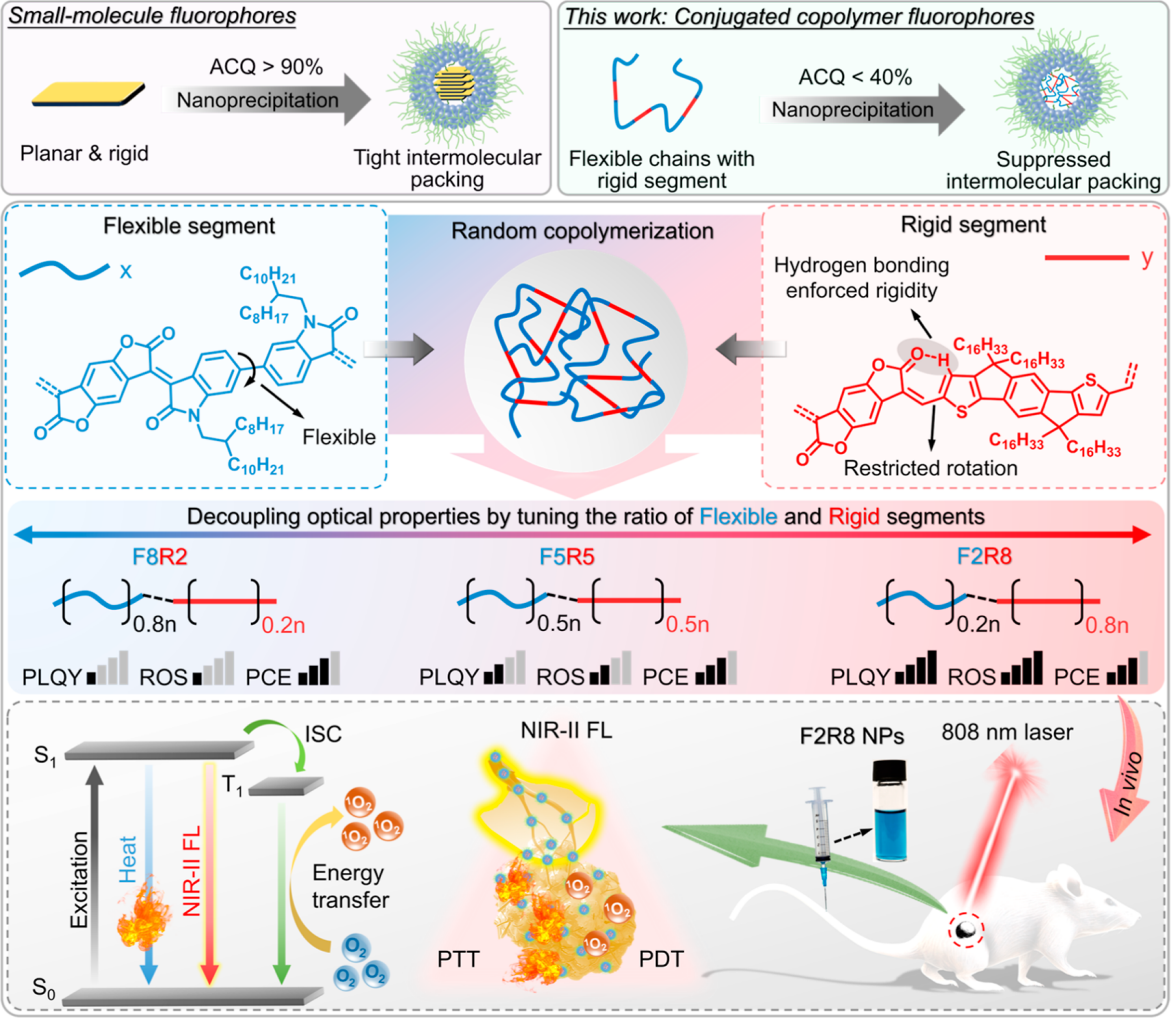
Schematic illustration of decoupling optical properties via ratio-tunable conjugated copolymers with hydrogen-bond-enforced rigidity for quenching-resistant NIR-II phototheranostics of cancer.

Conjugated polymers offer exceptional photostability, strong light-harvesting capability, and facile structural tunability.^32–40^ Yet, most reported polymeric phototheranostic agents are homopolymers consisting of a single repeating unit (Figure S6 and Table S1).^40–47^ Such homopolymer designs often couple absorption, emission, and energy dissipation within one structural motif, limiting the independent optimization of the NIR-II fluorescence, ROS production, and photothermal conversion. Moreover, homopolymers dominated by rigid chromophores can still suffer from strong interchain interactions and ACQ in nanoparticle assemblies, resulting in a suboptimal imaging brightness and therapeutic outcomes.^48^

Here, we introduce a copolymer modularity strategy to achieve quenching-resistant NIR-II phototheranostics by partially decoupling optical functions at the segment level, providing a distinct means of mitigating ACQ while enabling composition-dependent modulation with reduced trade-offs over fluorescence, photothermal, and photodynamic behaviors. Our copolymers integrate a flexible segment that disrupts excessive π–π packing together with a hydrogen-bond-enforced rigid segment that enhances conformational rigidity and promotes fluorescence emission and ROS generation. By precisely tuning the fraction of the rigid segment, we obtained four copolymers (F8R2, F5R5, F2R8, and R10), which provide a representative gradient in rigid-segment content for systematically probing composition–property relationships. Among them, F8R2, F5R5, and F2R8 were selected for detailed study due to their strong absorption in the NIR region (Figure S7).^49^ In contrast, R10 was not emphasized in the main text, as its absorption at 808 nm is comparatively negligible; however, under 750 nm excitation, R10 nanoparticles exhibit further enhanced ROS generation (∼1.8-fold higher than F2R8), supporting the positive correlation between rigid segment content and photodynamic performance. Increasing the rigid-segment content systematically improves NIR-II fluorescence and ROS generation while only slightly lowering photothermal conversion efficiency, demonstrating that copolymer composition offers an effective means of programming phototheranostic performance. Notably, even at a rigid-segment content of 80%, F2R8 retains more than 60% of its fluorescence in the aggregated nanoparticle state, together with strong ROS production, which represents a considerable improvement over conventional rigid small-molecule chromophores that typically lose more than 90% of their optical activity upon aggregation. Finally, the optimized copolymer nanoparticles enable high-contrast NIR-II fluorescence imaging-guided tumor phototherapy *in vivo*, demonstrating a generalizable route to overcome the long-standing “rigidity-ACQ” dilemma in organic NIR-II phototheranostics. Compared with existing NIR-II phototheranostic systems, this ratio-tunable copolymer strategy offers several advantages. By partially decoupling optical functions at the segment level, it enables simultaneous enhancement of fluorescence, ROS generation, and photothermal effects with reduced trade-offs, overcoming the intrinsic trade-offs in conventional small-molecule systems. The combination of flexible segments and hydrogen-bond-enforced rigid units suppresses π–π stacking while maintaining conformational rigidity, leading to improved resistance to aggregation-caused quenching and enhanced performance in nanoparticle states. In addition, the tunable composition allows composition-dependent modulation of photophysical properties within a single platform. Overall, these findings illustrate that segment-level copolymer design provides a practical and versatile approach for improving quenching resistance and enhancing the functional tunability of organic NIR-II imaging and therapeutic agents.

## 2 Results and Discussion

### 2.1 Synthesis and Theoretical Calculations of Copolymers

To be more specific, a flexible segment is constructed by linking 1,1′-bis­(2-octyldodecyl)-[6,6′-biindoline]-2,2′,3,3′-tetraone and 3,7-dihydrobenzo­[1,2-b:4,5-b’]­difuran-2,6-dione, while a rigid segment consists of 4,4,9,9-tetrahexadecyl-4,9-dihydro-s-indaceno­[1,2-b:5,6-b’]­dithiophene-2,7-dicarbaldehyde and 3,7-dihydrobenzo­[1,2-b:4,5-b’]­difuran-2,6 dione. Specifically, the flexible segment was chosen to effectively reduce intermolecular π–π interactions and alleviate ACQ, while the rigid segment was selected to enhance molecular planarity, promote radiative decay, and facilitate ROS generation. This design enables a balanced integration of structural flexibility and rigidity to optimize the photophysical and phototheranostic performance of the copolymers. Random copolymers F8R2, F5R5, F2R8, and R10 were prepared by systematically decreasing the fraction of flexible relative to rigid repeat units to modulate the macromolecular stiffness.^49^ The polymerization process lacks structural selectivity due to the similar reactivity of the monomer units and linker, resulting in a random copolymer. This structural disorder reduces segment packing and intermolecular π–π interactions, thereby alleviating aggregation-caused quenching effects. The polydispersity indices (PDIs) of the three copolymers are 2.65 for F8R2, 2.08 for F5R5, and 2.11 for F2R8, respectively. The relatively comparable PDI values indicate similar molecular weight distributions, thereby minimizing potential influences from polymer dispersity and supporting the reliability of the subsequent performance comparisons.

As illustrated in Figure 2A and E, the optimized ground-state (S_0_) geometry of the flexible segment exhibits a markedly twisted conformation, whereas the rigid segment maintains a planar, fully conjugated backbone. Quantum Theory of Atoms in Molecules (QTAIM) was employed to evaluate the O···H hydrogen-bond binding energy (BE).^50^ In the rigid segment, the calculated BE of −3.82 kcal/mol corresponds to a weak-to-moderate hydrogen bond that stabilizes the planar conformation and effectively locks the only rotatable single bond between two units, thereby suppressing internal rotation. Together with the fully fused-ring backbone, this interaction enforces overall conformational rigidity. In comparison, the flexible segment also contains an O···H hydrogen bond with a slightly higher BE of −4.41 kcal/mol, indicative of a moderate interaction. This hydrogen bond is located across a C=C double bond, which is intrinsically nonrotatable and thus locally rigidifies this subunit. However, this hydrogen-bonded moiety is connected to another segment through a C–C single bond that remains freely rotatable and is not constrained by the hydrogen bond. Consequently, despite comparable hydrogen-bond strength, the flexible segment retains overall conformational freedom. This comparison highlights that the distinct photophysical behaviors arise not simply from hydrogen-bond strength but from how effectively the hydrogen bond restricts rotation at the molecular level.

**Figure 2:**
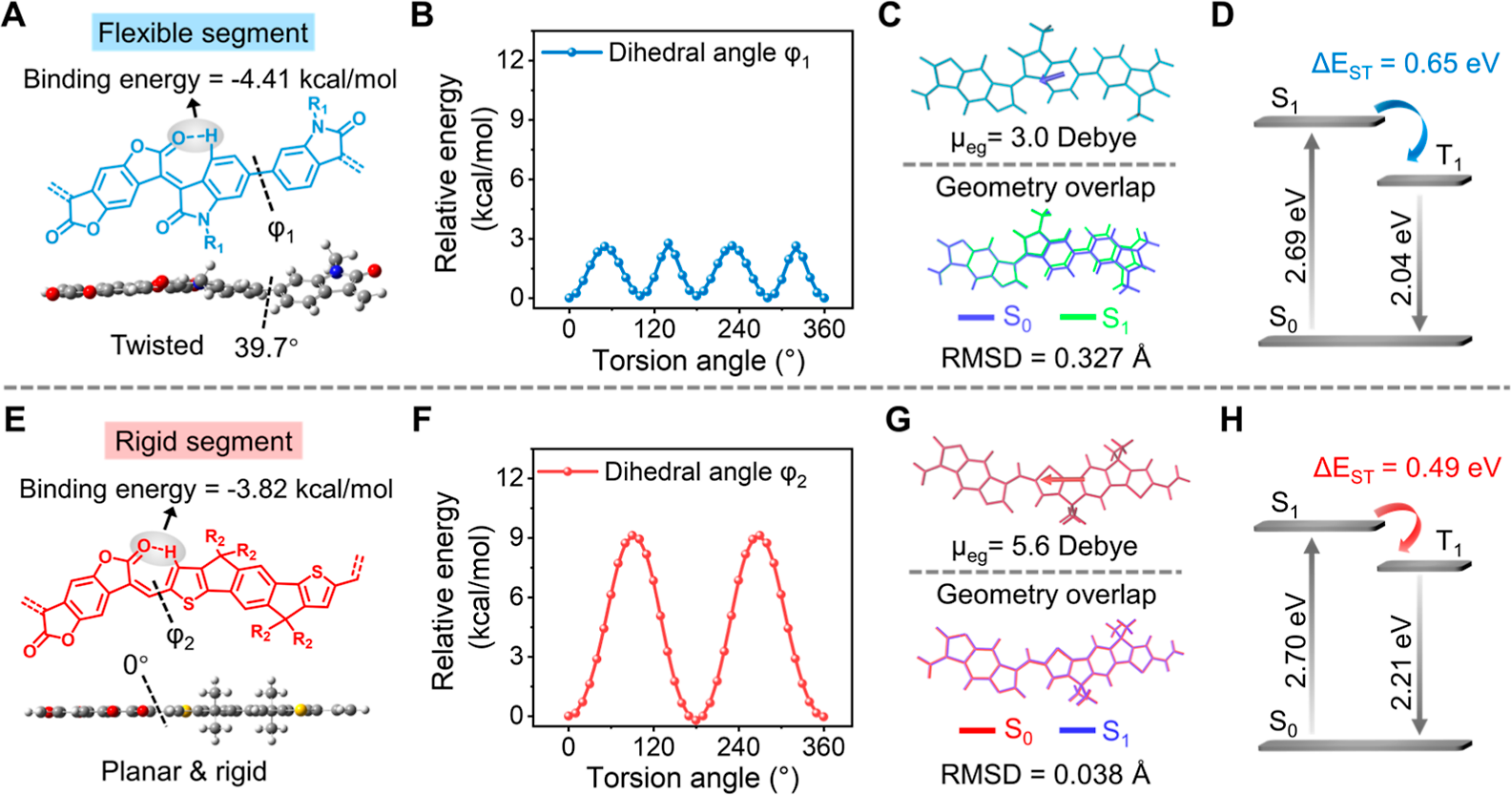
DFT calculations of flexible and rigid segments at the M06–2*X*/6–311G­(d) level. (A) Geometry optimization of the twisted flexible segment and hydrogen-bond binding energy calculations. (B) Dihedral angle potential energy scans of the flexible segment. (C) Transition dipole moment, ground/excited-state geometries, and RMSD values of the flexible segment. (D) Energy-level diagram of the lowest excited singlet (S_1_) and lowest triplet (T_1_) states of the flexible segment with Δ*E*_ST_ representing the S_1_-T_1_ energy gap. (E) Geometry optimization of the planar rigid segment and hydrogen-bond binding energy calculations. (F) Dihedral angle potential energy scans of the rigid segment. (G) Transition dipole moment, ground/excited-state geometries, and RMSD values of the rigid segment. (H) Energy-level diagram of the S_1_ and T_1_ states of the flexible segment with Δ*E*_ST_.

To further assess the flexibility of the designed repeat units, relaxed dihedral scans were carried out beginning from their lowest energy geometries with alkyl side chains replaced by methyl groups to reduce computational cost. Calculations were performed using Gaussian 16 at the M06–2*X*/6–31G­(d) level.^51^ As shown in Figure 2B, the flexible segment exhibits energy minima at 0°, 100°, and 180°, with a rotational barrier of around 2.7 kcal/mol, indicating substantial conformational freedom and a high propensity for thermally induced twisting. By contrast, the rigid segment (Figure 2F) shows stable minima only at 0° and 180°, accompanied by a much higher rotational barrier of 9.1 kcal/mol, confirming significantly restricted torsional motion and a predominantly planar backbone.^52^ Such rigidity is expected to contribute to enhanced fluorescence emission and ROS generation.^53–55^

Given that both the absorption cross-section and radiative rate constant are proportional to the square of the transition dipole moment (μ_*eg*_), a larger μ_*eg*_ typically results in stronger light–fluorophore interactions and enhanced fluorescence.^56^ As shown in Figure 2C and G, the rigid segment demonstrates a substantially greater μ_*eg*_ than the flexible segment, providing more favorable channels for efficient fluorescence. Analysis of the optimized S_0_ and the lowest excited singlet (S_1_) geometries further reveals that the flexible segment undergoes pronounced structural rearrangement upon excitation, indicated by a large root-mean-square displacement (RMSD) of 0.327 Å (Figure 2C). In contrast, the rigid segment shows a minimal RMSD of 0.038 Å (Figure 2G), suggesting very limited excited-state distortion and therefore a reduced nonradiative decay pathway.^15^

Photophysical evaluation of the singlet–triplet energy gap (Δ*E*_ST_) provides additional insights into excited-state dynamics relevant to photodynamic therapy.^57,58^ As shown in Figure 2D and H, the flexible segment shows a relatively large Δ*E*_ST_ of 0.65 eV, while the rigid segment exhibits a markedly smaller Δ*E*_ST_ of 0.49 eV. This variation is predominantly attributed to the enhanced rigidity and planarization in the rigid segment, which modulate frontier molecular orbital overlap. The reduced Δ*E*_ST_ in the rigid segment suggests more efficient intersystem crossing (ISC) and triplet-state population, thereby facilitating photodynamic activity.

Collectively, these findings highlight the pivotal role of structural rigidity in modulating excited-state energetics and underscore its importance in the rational design of copolymer systems with an optimized photophysical and photodynamic performance.

### 2.2 Photophysical Properties of Copolymer Nanoparticles

Water-dispersible NPs of F8R2, F5R5, and F2R8 were prepared by nanoprecipitation for subsequent biomedical applications. In the presence of the amphiphilic Pluronic F127, the copolymers coassemble with F127 to form colloidally stable nanoparticles. The resulting nanoparticles exhibit an average hydrodynamic diameter of ∼60 nm, as determined by dynamic light scattering (DLS) (Figure 3A). Transmission electron microscopy (TEM) further confirms their spherical morphology and reveals particle sizes consistent with the DLS results (Figure S8). The size distributions exhibited only minor fluctuations over 14 days in aqueous dispersion, phosphate-buffered saline (PBS), and 10% fetal bovine serum (FBS) indicating good colloidal stability under different conditions (Figure S9). Zeta potentials of −18.4, −22.1, and −14.2 mV were measured for the three NP formulations (Figure S10), which are attributed to the negatively charged nature of the F127 polymer. Molar extinction coefficients of F8R2, F5R5, and F2R8 NPs were also determined. As illustrated in Figures 3B and S11, all three copolymer NPs exhibit strong absorption in the NIR region, underscoring their potential in phototheranostics. Correspondingly, the NP emissions extend beyond 1100 nm (Figure 3C), confirming photophysical properties suitable for biological imaging within the NIR-II window.

**Figure 3:**
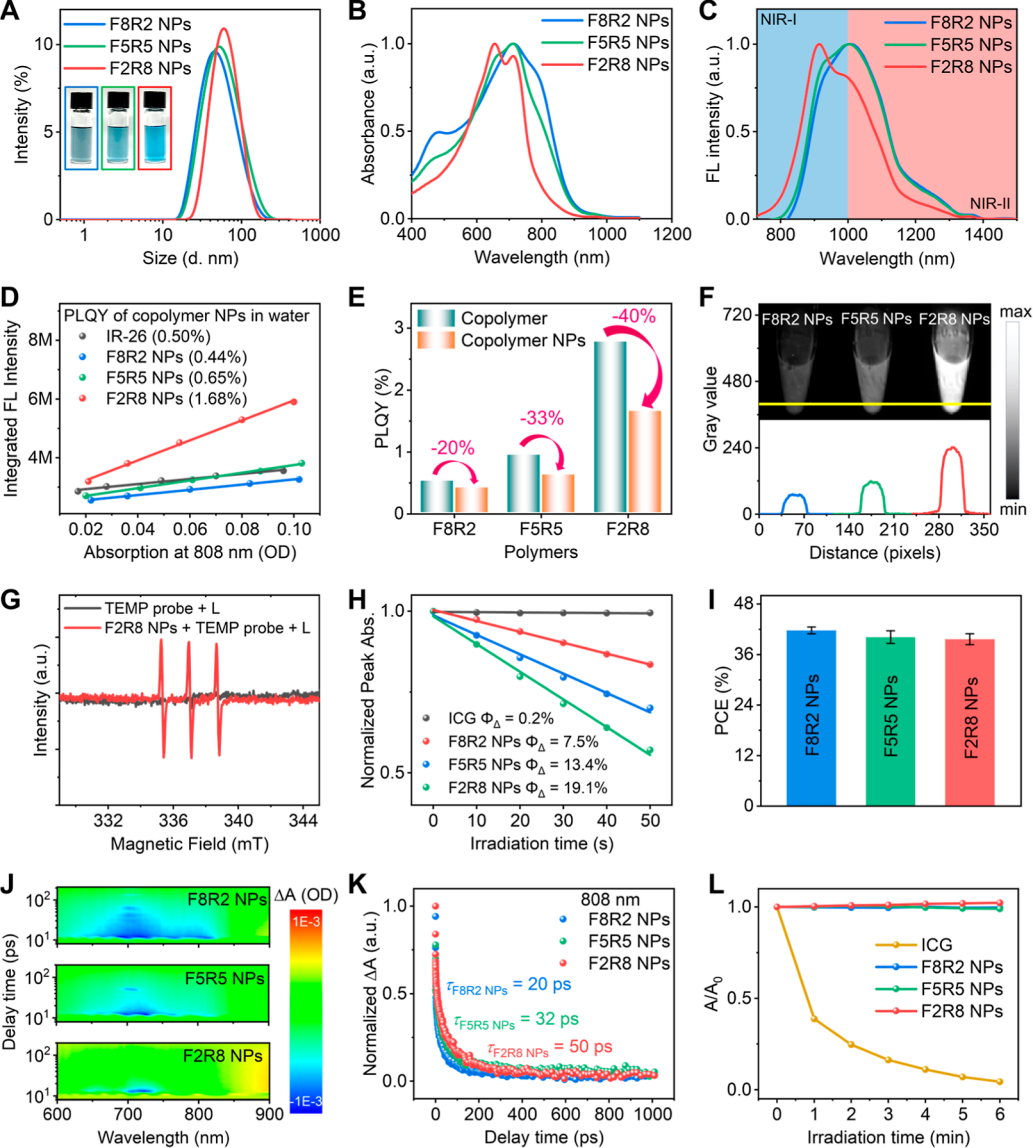
Colloidal and photophysical properties of aqueous NP dispersions of F8R2, F5R5, and F2R8. (A) Size distribution of F8R2, F5R5, and F2R8 NPs. Insets: photographs of their aqueous dispersions. (B) UV–vis–NIR absorption spectra of F8R2, F5R5, and F2R8 NP dispersions. (C) Normalized fluorescence spectra of F8R2, F5R5, and F2R8 NP dispersions. (D) Integrated NIR-II fluorescence intensities (900–1500 nm) of F8R2, F5R5, and F2R8 NPs in water at five concentrations using IR-26 in DCE as a reference. (E) Comparison of calculated PLQYs for the copolymers in THF versus their NPs in water. (F) NIR-II fluorescence imaging of the three NP dispersions (50 μg/mL) under 690 nm laser excitation, with fluorescence intensity profiles extracted along the indicated yellow line. (G) EPR spectra of the TEMP probe with/without F2R8 NPs after 5 min irradiation of 808 nm laser (500 mW/cm^2^). (H) Linear fitting of quantitative singlet oxygen detection by monitoring relative DCFH fluorescence intensities of the three NPs under 808 nm excitation (power density: 330 mW/cm^2^) at different time points. (I) Quantitative PCE values with error bars for all three NPs: (41.7 ± 0.8)% for F8R2 NPs, (40.1 ± 1.5)% for F5R5 NPs, and (39.6 ± 1.3)% for F2R8 NPs. (J) 2D pseudocolor fs-TA spectra of F8R2 NPs, F5R5 NPs, and F2R8 NPs (50 μg/mL). Excitation: 700 nm pump pulse. (K) Normalized kinetic curve of F8R2 NPs, F5R5 NPs, and F2R8 NPs at 808 nm within the GSB region, fitted with a single-exponential decay. (L) Relative absorbance at peak sites illustrating photostabilities of F8R2, F5R5, and F2R8 NPs and the commercial fluorophore ICG.

Measured photophysical properties are summarized in Table S2. Relative photoluminescence quantum yields (PLQYs) of copolymer solutions and their corresponding NPs were quantified using IR-26 (PLQY = 0.50% in 1,2-dichloroethane (DCE)) as the standard reference (Figures S12 and S13).^59^ As depicted in Figures S12I, 3D, and E, the PLQYs decreased by approximately 20–40% upon transitioning from THF solutions to aqueous NPs for all three copolymers, suggesting attenuated intermolecular interactions and improved resistance to ACQ. This behavior can be attributed to its intrinsic structural characteristics. In the nanoparticle state, polymer chain entanglement hinders dense π–π stacking between rigid segments, resulting in less compact packing and increased intermolecular distances. Meanwhile, the incorporation of flexible units further disrupts ordered molecular packing and effectively reduces intermolecular π–π interactions. Together, these factors suppress detrimental intermolecular electronic coupling and mitigate ACQ, thereby preserving emission efficiency in the aggregated state. To further elucidate aggregation effects, fluorescence spectra were recorded in mixed organic solvent/water systems (Figure S14). As the water fraction (*f*_w_, vol %) increased, the emission of the molecularly dissolved species was progressively quenched, consistent with ACQ. Notably, F2R8 NPs demonstrated a relatively high PLQY of 1.68% in aqueous solution. This pronounced anti-quenching behavior is attributed to inhibited intermolecular packing in the aggregated state, which alleviates aggregation-associated nonradiative dissipation and favors radiative deactivation. Fluorescence imaging (FLI) of NP dispersions (Figure 3F) further shows that incorporation of rigid segments markedly enhances emissive output.

The generation of reactive oxygen species (ROS), including singlet oxygen (^1^O_2_), superoxide radicals (O_2_^•–^), and hydroxyl radicals (•OH), was systematically evaluated.^60,61^ Upon irradiation in the presence of the ^1^O_2_ probe 1,3-diphenylisobenzofuran (DPBF), a pronounced decrease of the 415 nm absorption band was observed, relative to the blank control (Figure S15), indicating efficient ^1^O_2_ production of polymers in THF. In contrast, under laser irradiation, neither dihydrorhodamine-123 (DHR123) and hydroxyphenyl fluorescein (HPF) showed measurable fluorescence enhancement (Figures S16 and S17), suggesting minimal production of O_2_^•–^ and •OH of polymers in THF. Using 2′,7′-dichlorodihydrofluorescein (DCFH) as a general ROS probe, F2R8 NPs produced a substantial fluorescence increase (Figure S18) approximately 8-fold higher than that of F8R2 NPs, highlighting the enhanced photodynamic efficacy conferred by the rigid segment. As shown in Figure 3G, the 2,2,6,6-tetramethylpiperidine (TEMP) probe alone does not exhibit any detectable electron paramagnetic resonance (EPR) signal under 808 nm laser irradiation. In contrast, the F2R8 NPs in the presence of TEMP display a characteristic three-line EPR signal upon 808 nm excitation, indicating the generation of singlet oxygen (^1^O_2_), which reacts with TEMP to form the corresponding TEMPO radical. The singlet oxygen quantum yields (Φ_Δ_) of the developed NPs were evaluated using DPBF as a trapping probe and ICG as a reference (Φ_Δ_ = 0.2%).^62^ As shown in Figure S19, all copolymer nanoparticles exhibit substantially higher singlet-oxygen generation efficiencies compared to ICG. Importantly, enhancement of Φ_Δ_s observed under 808 nm irradiation is consistent with that measured under 750 nm excitation, underscoring the robustness of their photodynamic performance across different near-infrared wavelengths. As further illustrated in Figure 3H, the Φ_Δ_ of F2R8 NPs (19.1%) is approximately 2.5-fold higher than that of F8R2 NPs (7.5%), highlighting the positive correlation between rigid segment content and ROS generation efficiency.

Photothermal characteristics of the copolymer NPs were evaluated under 808 nm laser irradiation (1 W/cm^2^). The photothermal conversion efficiency (PCE, η) was determined by a standard heating–cooling method (Figures 3I, S20, and S21; Tables S3–S5).^63,64^ As shown in Figure S21D, temperature elevations for F2R8 NP dispersions at varying concentrations during 300 s of irradiation were demonstrated. Notably, a dispersion containing 150 μg/mL of F2R8 NPs exhibited a temperature rise exceeding 45 °C following 5 min irradiation, demonstrating good photothermal performance. The thermal response remained consistent over five on/off irradiation cycles (Figure S22), indicating excellent photothermal stability and supporting their suitability for biomedical applications.^65,66^

To provide further mechanistic insights, we briefly correlate the theoretical results (Figure 2) with the experimental observations (Figure 3). The calculations reveal that hydrogen-bond-enforced rigidity in the designed segments leads to increased rotational energy barriers and minimal conformational changes between the S_0_ and S_1_ states, indicating suppressed intramolecular motion. In addition, the reduced Δ*E*_ST_ of the rigid segment suggests more favorable intersystem crossing. These features collectively contribute to enhanced fluorescence emission and ROS generation at the molecular level. Consistent with these predictions, the experimental results (Figure 3) show that increasing the rigid-segment fraction leads to a gradual increase in both fluorescence quantum yield and ROS generation efficiency while only slightly affecting photothermal conversion. This agreement between the theory and experiment demonstrates that hydrogen-bond-enforced rigidity effectively modulates excited-state relaxation pathways and that ratio-tunable copolymer design enables composition-dependent adjustment of energy dissipation processes with reduced trade-offs, thereby achieving partial decoupling to a significant extent of fluorescence, photodynamic, and photothermal functions.

Femtosecond transient absorption (fs-TA) spectroscopy was employed to probe ultrafast excited-state dynamics. Pseudocolor fs-TA maps (Figure 3J) exhibit a clear ground-state bleaching (GSB), reflecting depletion of ground-state population upon photoexcitation and thus tracking excited-state kinetics.^67–69^ As shown in Figure 3K, the fastest decay component for F8R2 NPs (∼20 ps) is assigned to an accelerated nonradiative relaxation channel, consistent with the higher PCE where excitation energy is efficiently dissipated as heat. In contrast, F2R8 NPs exhibit more prolonged decay kinetics, aligning with their superior ROS generation and elevated PLQY. These results suggest that suppressing nonradiative relaxation extends the excited-state lifetime, thereby facilitating either ISC to triplet manifolds for ROS production or radiative recombination for fluorescence emission. Collectively, the mechanistic picture provides a rational basis for incorporating rigid segments in copolymers to optimize synergistic photothermal, photodynamic, and fluorescence performance.

Comparative absorption studies (Figures S23 and 3L) show that the copolymer nanoparticles exhibit significantly enhanced photostability relative to indocyanine green (ICG) under continuous 808 nm laser irradiation (0.5 W cm^–2^). The nanoparticles retain nearly their initial absorbance after 6 min, whereas ICG undergoes pronounced photobleaching. Consistent with this trend, fluorescence spectra measurements (Figure S24) reveal that all copolymer nanoparticles maintain stable emission under identical conditions, while the fluorescence intensity of ICG decreases sharply. These results together demonstrate the superior photostability and robustness of the copolymer system. To further evaluate the role of hydrogen bonding in the aggregated state, dimethyl sulfoxide (DMSO), a known hydrogen-bond disruptor, was introduced into the aqueous dispersions of the copolymer nanoparticles.^70,71^ As shown in Figure S25, the addition of 16.7% DMSO results in negligible changes in both the absorption and fluorescence spectra for all three copolymer nanoparticles. This observation suggests that the disruption of hydrogen bonding by DMSO is limited under these conditions. A plausible explanation is that the copolymer cores are effectively shielded by the F127 matrix, which restricts the penetration of DMSO into the nanoparticle interior and thus suppresses its interaction with the copolymer chains. Consequently, the intrinsic hydrogen-bonding interactions within the rigid segments remain largely preserved, maintaining the photophysical properties of the nanoparticles.

To further elucidate the origin of the observed decoupling behavior, the radiative (*k*_r_) and nonradiative (*k*_nr_) decay rates were estimated from the PLQY and fluorescence lifetime (τ, Figure S26) using *k*_r_ = PLQY/τ and *k*_nr_ = (1–PLQY)/τ. As summarized in Table S6, *k*_r_ increases significantly from F8R2 to F2R8, whereas *k*_nr_ remains on the order of 10^9^ s^–1^ with only minor variation. Notably, *k*_nr_ represents the sum of all nonradiative pathways, including internal conversion (k_IC_) and intersystem crossing (k_ISC_). Combined with the progressively enhanced ROS generation, these results suggest an increased contribution of k_ISC_ accompanied by a corresponding decrease in k_IC_ along the series. This internal redistribution of nonradiative decay channels provides a clear explanation for the observed performance trend: despite a substantial enhancement in fluorescence and ROS generation, the photothermal conversion efficiency shows only a slight decrease. Therefore, the energy dissipation is not simply reduced but is rebalanced between competing pathways, enabling simultaneous optimization of radiative and photodynamic processes while largely preserving photothermal output.

### 2.3 Anti-quenching Behavior

As illustrated in Figures 4A–C and S1–S5, planar organic small molecules typically experience substantial π–π stacking upon aggregation, which greatly promotes nonradiative decay and leads to severe ACQ. In many luminophores, fluorescence quenching exceeds 90%, representing a major limitation in the design of fluorescent organic materials. In contrast, Figure 3E reveals that the transition of the copolymers from molecular dissolved states in THF to NPs in aqueous media leads to a PLQY reduction of approximately 20–40%. A trade-off is observed with increasing rigid-segment content, where enhanced fluorescence is accompanied by moderately increased ACQ. This is balanced by the incorporation of flexible segments, which partially disrupt π–π stacking. As a result, the overall fluorescence quantum yield in the aggregated state still increases, accompanied by enhanced ROS generation, leading to improved phototheranostic performance. A comparison of quenching behaviors among five representative planar organic small molecules and the three developed copolymers is summarized in Figure 4D. The comparatively modest quenching (20–40%) indicates that the copolymers experience significantly attenuated intermolecular interactions in their aggregated states and thereby demonstrating enhanced resistance to ACQ.

**Figure 4:**
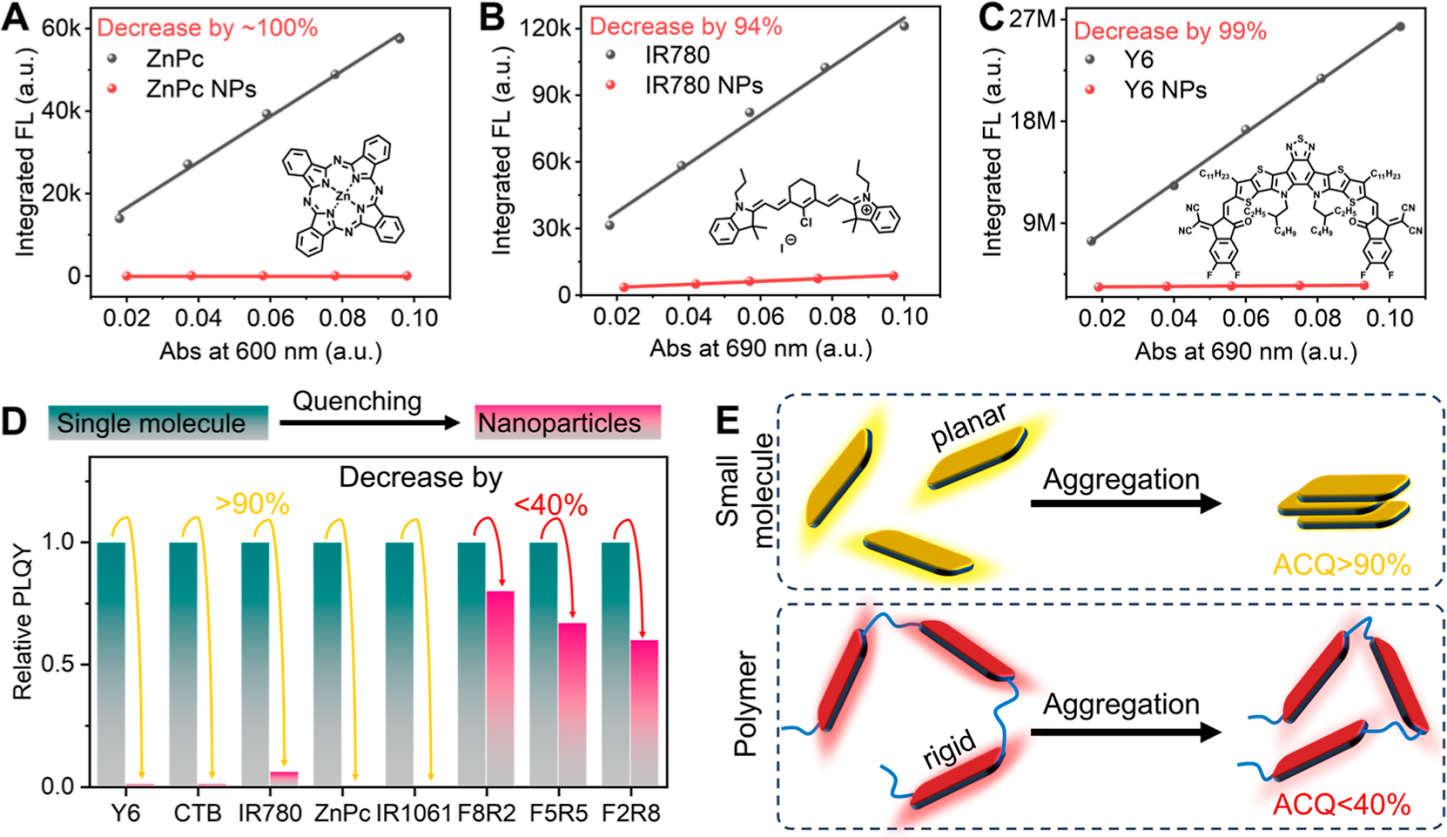
Fluorescence ACQ behaviors of the obtained copolymers and typical planar small molecules. (A) Plots of integrated fluorescence intensities of ZnPc and ZnPc NPs at five concentrations. (B) Plots of integrated fluorescence intensities of IR780 and IR780 NPs at five concentrations. (C) Plots of integrated fluorescence intensities of Y6 and Y6 NPs at five concentrations. (D) Comparison of ACQ behaviors between typical planar small molecules and the obtained copolymers. (E) Scheme of quenching behaviors of obtained copolymers versus typical planar small molecules.

Figure 4E depicts the distinct aggregation behaviors of planar organic small molecules versus the copolymer system. The amphiphilic F127 plays a key role in regulating aggregation behavior. In copolymer systems, the polymer chains are confined within the micellar core and, due to their covalent connectivity and segmental heterogeneity, are unable to reorganize into tightly π–π stacked structures. In contrast, small-molecule dyes can readily rearrange and pack into ordered aggregates even within the confined environment, leading to stronger aggregation-induced quenching. Planar small molecules typically exhibit strong fluorescence in their isolated molecular state due to efficient radiative transitions. However, upon aggregation, extensive π–π stacking facilitates nonradiative decay pathways, drastically reducing fluorescence intensity.^72^ The copolymers, particularly those incorporating a high proportion of rigid segments, show markedly increased PLQYs in dilute solution, resembling the intense emission of planar small molecules in their monomeric form. This enhancement stems from suppressed nonradiative relaxation enabled by backbone rigidification. Notably, even F2R8 containing as high as 80% rigid segments does not exhibit severe fluorescence quenching upon aggregation, with the quenching level remaining below 40%, which is substantially lower than that observed for conventional planar small molecules. This exceptional suppression of ACQ suggests that the covalent incorporation of flexible and rigid segments enforces spatial separation between emissive units, thereby reducing excessive aggregation and limiting nonradiative decay. Such a molecular design strategy offers a generalizable approach to minimizing ACQ in organic aggregates for biomedical applications.

### 2.4 *In Vitr*o Phototherapeutic Performance

Comparative *in vitro* evaluation of the three copolymer NPs (Figure 5A) reveals that F2R8 NPs exhibit elevated ROS generation and enhanced PDT efficacy. CCK-8 viability assays demonstrated that, under low-power 808 nm laser irradiation (0.33 W/cm^2^, insufficient to induce thermal effects over 43 °C), F2R8 NPs induce greater cytotoxicity relative to the other copolymer NPs, confirming their improved PDT efficacy. Owing to their enhanced ROS-generating capacity, F2R8 NPs were selected for further evaluation of combined photodynamic and photothermal effects in cancer cells. As illustrated in Figure 5B, treatment of 4T1 cells with 60 μg/mL F2R8 NPs followed by 808 nm laser irradiation (1 W/cm^2^) reduces cell viability to approximately 12%. In contrast, F2R8 NPs without irradiation produce negligible cytotoxicity, demonstrating that the therapeutic effect arises from photoactivation rather than intrinsic NP toxicity. Moreover, dark-condition cytotoxicity studies in 3T3 normal cells across NP concentrations up to 100 μg/mL show no detectable toxicity, confirming the excellent biocompatibility of F2R8 NPs (Figure 5C). Overall, these results highlight the high efficacy of F2R8 NPs as NIR-activated phototherapeutic agents with minimal dark toxicity.

**Figure 5:**
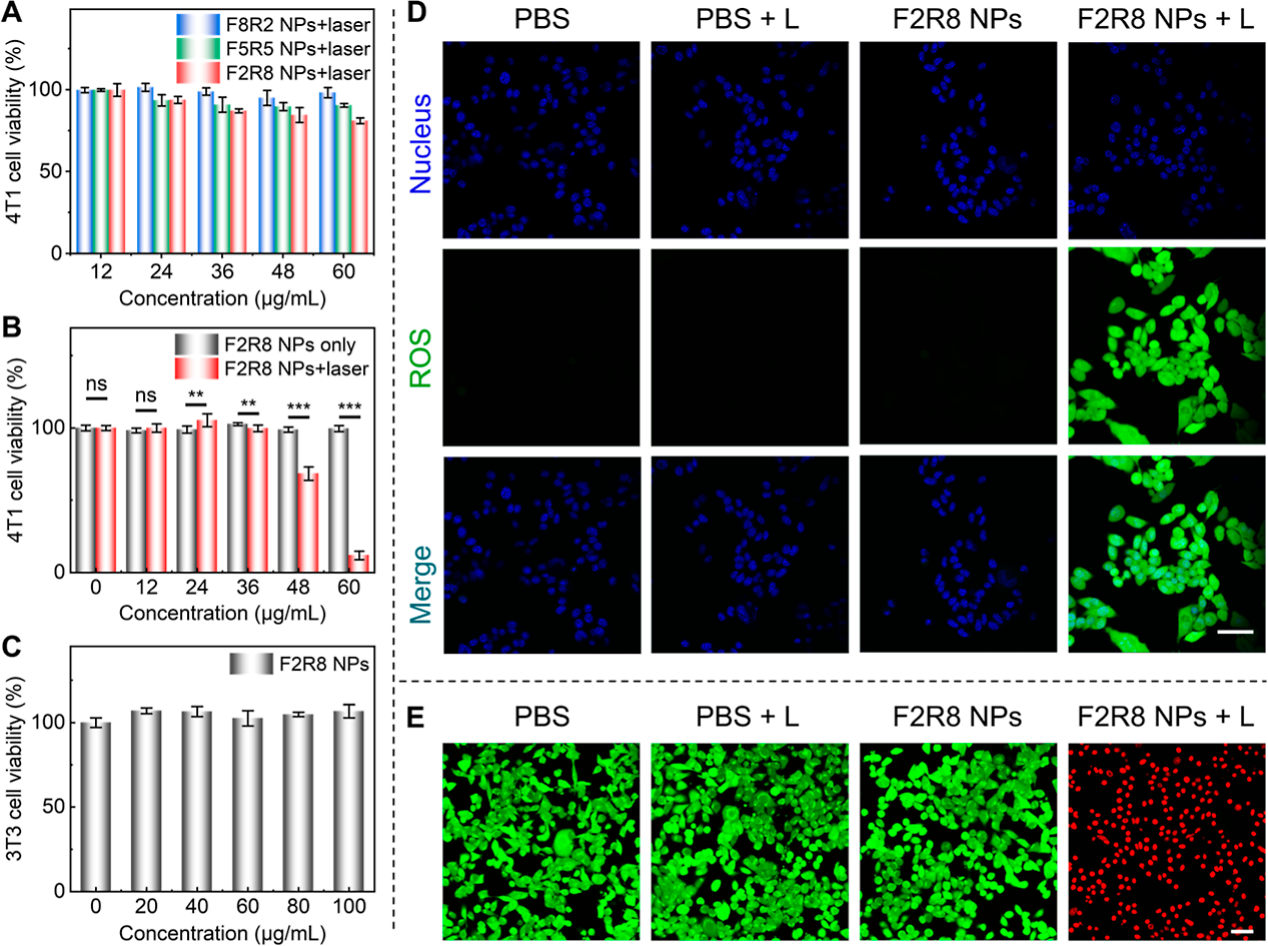
*In vitro* phototherapeutic performance of F2R8 NPs. (A) Cell viability of 4T1 cells treated with varying concentrations of three copolymer NPs with 808 nm laser irradiation (0.33 W/cm^2^, 15 min). Data are presented as mean ± standard deviation (*n* = 4). (B) Cell viability of 4T1 cells treated with varying concentrations of F2R8 NPs with or without 808 nm laser irradiation (1 W/cm^2^, 5 min). Data are presented as mean ± standard deviation (*n* = 4). **p < 0.05; ***p < 0.001; ns, no significant differences. (C) Dark toxicity of 3T3 cells incubated with F2R8 NPs at different concentrations for 24 h. Data are presented as mean ± standard deviation (*n* = 4). (D) Confocal fluorescence images of intracellular ROS generation in 4T1 cells under various treatment conditions. DCFH-DA was used as the ROS probe and Hoechst 33,342 as the nuclear staining. Scale bar: 50 μm. (E) Live–dead cell imaging of 4T1 cells under various treatment conditions, stained with calcein-AM and ethidium homodimer-1. Scale bar: 50 μm.

Intracellular ROS generation was further assessed using DCFH-DA, with Hoechst 33,342 counterstaining for nuclear visualization. As shown in Figure 5D, no detectable green fluorescence indicative of oxidized DCF is observed in PBS, PBS + L, or F2R8 NP-only groups. In contrast, 4T1 cancer cells incubated with F2R8 NPs and subjected to laser irradiation exhibit a strong green fluorescence, confirming efficient intracellular ROS production. Additional cellular uptake experiments were performed using confocal microscopy of Cy3-labeled nanoparticles, and the resulting images were merged with ROS detection to clearly illustrate intracellular localization (Figure S27). Cy3 fluorescence within F2R8 nanoparticles (formulated from F127 and DSPE-PEG2000-Cy3 at a 9:1 mass ratio) was used to track nanoparticle distribution. The results confirm efficient cellular internalization and uniform intracellular distribution of the nanoparticles, providing a suitable platform for efficient ROS generation and subsequent tumor cell ablation. The potent phototherapeutic effect of F2R8 NPs was further validated by using live/dead staining. Viable cells in the PBS, PBS + L, and F2R8 NP groups exhibit strong green cytoplasmic fluorescence from calcein AM, whereas the F2R8 NPs + L group shows prominent red nuclear fluorescence from ethidium homodimer-1, indicative of cell death (Figure 5E). These results conclusively demonstrate the strong combined photodynamic and photothermal anticancer efficacy of the F2R8 NPs.

### 2.5 *In Vivo* NIR-II Fluorescence Imaging and Phototherapeutic Efficacy

Figure 6A and B illustrate visualization of murine vasculature following tail intravenous administration of F2R8 NPs under long-pass (LP) filters of 900 and 1100 nm. With increasing LP cutoff, vascular contrast is progressively enhanced while background autofluorescence diminishes. Quantitative analysis of cross-sectional intensity profiles reveals a decrease in full width at half-maximum (FWHM) from 6.43 pixels at 900 nm to 5.78 pixels at 1100 nm, demonstrating the improved spatial resolution achievable in the NIR-II imaging window. As shown in Figure S28, mice were intravenously injected with F2R8 NPs, IR780 NPs, or IR1061 NPs at the same dosage, followed by immediate whole-body vascular imaging using an NIR-II imaging system with a 900 nm long-pass filter. Notably, the F2R8 NP-treated group enables clear visualization of vascular structures with a narrow FWHM of 5.29 pixels in the hindlimb vessels. In contrast, IR780 NPs exhibit a significantly broader FWHM (9.69 pixels), indicating inferior spatial resolution, while IR1061 NPs fail to provide discernible vascular imaging, showing only weak fluorescence signals localized in major organs. These differences are mainly attributed to the severe ACQ effect of cyanine-based dyes. Collectively, these results demonstrate that F2R8 NPs possess markedly enhanced NIR-II fluorescence brightness and imaging performance *in vivo*.

**Figure 6:**
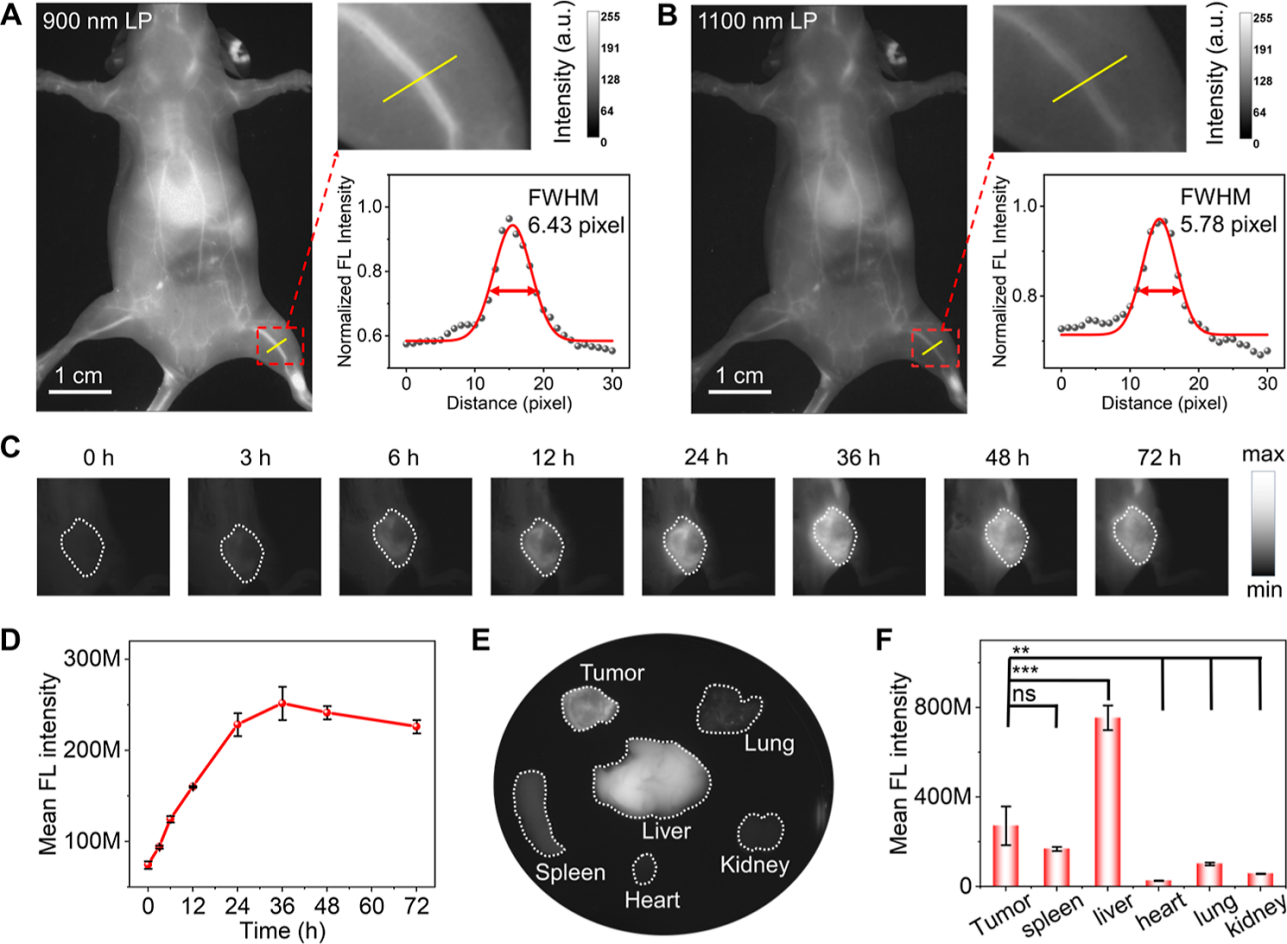
*In vivo* NIR-II fluorescence imaging of whole-body vasculature and tumor accumulation of F2R8 NPs. (A) Whole-body vascular imaging acquired using a 900 nm LP filter with the corresponding FWHM analysis. (B) Whole-body vascular imaging acquired using a 1100 nm LP filter with corresponding FWHM analysis. (C) NIR-II FLI images of tumor-bearing mice at various time points following intravenous administration of F2R8 NPs (900 nm LP filter). (D) Relative fluorescence intensities of the tumor site post-injection of F2R8 NPs (*n* = 3). (E) *Ex vivo* NIR-II FLI images of tumors and major organs collected 36 h postinjection of F2R8 NPs. (F) Fluorescence intensities of tumors and major organs collected 36 h post-injection of F2R8 NPs. **p < 0.05; ***p < 0.001; ns, no significant differences.

As shown in Figure 6C, NIR-II fluorescence images of 4T1 tumors collected at varied time points postinjection exhibit gradually increasing signal intensity. The fluorescence intensity reached its maximum at 36 h (Figure 6D), which was used as the optimal therapeutic time point. *Ex vivo* fluorescence imaging of major organs and tumor tissues harvested at 36 h (Figure 6E,F) reveals predominant accumulation in the liver and tumor tissue, confirming effective tumor targeting of F2R8 NPs.

To assess *in vivo* phototherapeutic performance, 4T1 tumor-bearing mice were irradiated with an 808 nm laser 36 h post-injection of F2R8 NPs. Infrared thermal imaging shows minimal temperature elevation in the PBS + L group, whereas the F2R8 NPs + L group reaches 57 °C within 5 min of laser irradiation (1 W/cm^2^), indicating efficient photothermal conversion (Figure 7A and B). Tumor growth curves (Figures S29, 7C–E) show that mice treated with F2R8 NPs followed by irradiation experience significant tumor ablation throughout the treatment period. In contrast, the PBS, PBS + L, and F2R8 NP-only groups display negligible tumor inhibition. Body-weight monitoring (Figure S30) indicates mild weight gain across all groups, supporting the biosafety of the treatment.

**Figure 7:**
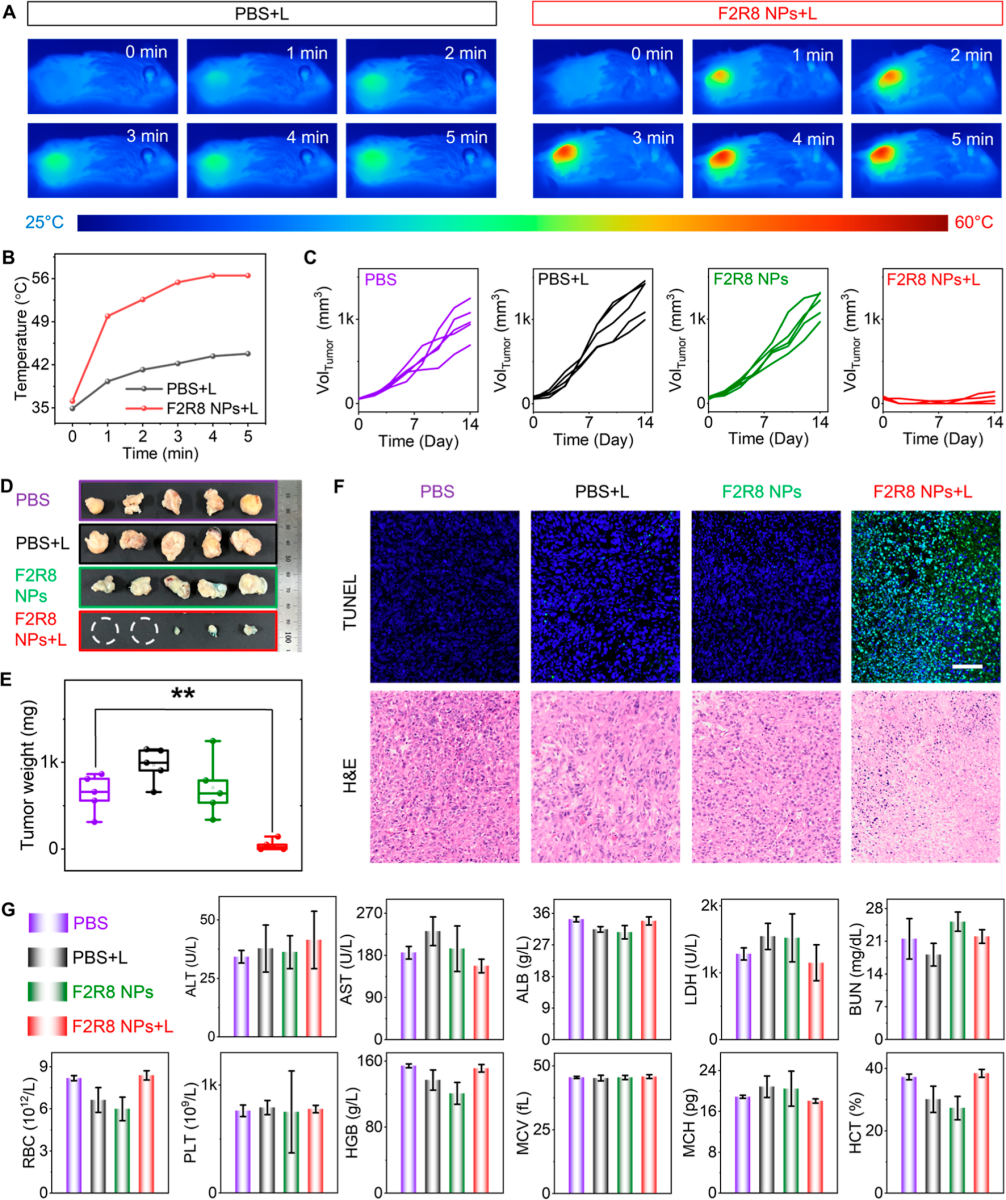
*In vivo* NIR-II FLI, anticancer performance and biocompatibility evaluation of F2R8 NPs. (A) Infrared thermal images of tumor-bearing mice under 808 nm laser irradiation (1 W/cm^2^) for 5 min in PBS + L and F2R8 NPs + L groups. (B) Corresponding temperature changes in the tumor region during irradiation. (C) Tumor volume progression in different treatment groups over a 14-day period (*n* = 5). (D) Photographs of excised tumors from each treatment group (*n* = 5). (E) Tumor weights of mice in various treatment groups (*n* = 5). (F) Histological analysis of tumor tissues using H&E staining and TUNEL assays in various treatment groups. Scale bar: 100 μm. (G) Blood biochemical and hematological analyses of mice after 14 days of treatment. Data are presented as mean ± standard deviation (*n* = 5).

Histological and hematological assessments further validated the therapeutic outcomes. Terminal deoxynucleotidyl transferase dUTP nick end labeling (TUNEL) staining (Figure 7F) confirms extensive apoptotic damage in tumors from the F2R8 NPs + L group. Hematoxylin and eosin (H&E) staining of tumor tissues reveals pronounced cellular destruction in this group. In contrast, no significant pathological abnormalities are observed in the control groups, although slight reductions in nuclear density can be noted in the PBS + L and NP-only groups at 24 h post-treatment, suggesting minor and transient cellular responses. However, H&E staining at this early time point mainly reflects short-term morphological changes rather than sustained inhibition of tumor proliferation. To further clarify this, Ki67 immunohistochemical staining was performed on the tumor sections collected at the same time point. As shown in Figure S31, Ki67 expression in the PBS + L and NP-only groups is comparable to that in the PBS group, indicating that proliferative activity is not significantly affected. This observation is consistent with the similar tumor growth profiles observed across these groups. H&E staining of major organs (heart, liver, spleen, lung, and kidney) shows a normal tissue morphology across all groups (Figure S32), suggesting minimal systemic toxicity.^73,74^ Blood biochemistry and complete blood panel analyses collected 14 days post-treatment show no significant differences between treated and control groups (Figure 7G), confirming the absence of hepatotoxicity, nephrotoxicity, and other systemic adverse effects.

### 2.6 Study Limitations

Despite these promising results, several limitations should be noted. The structure–property relationships in multicomponent copolymers are inherently more complex than those of small-molecule systems, which may increase synthetic and characterization challenges and could pose additional considerations for scalable synthesis and reproducibility toward clinical translation. In addition, although the copolymer design enables effective modulation of multiple optical functions, complete decoupling of fluorescence, ROS generation, and photothermal processes remains difficult due to residual interchain interactions.

Furthermore, while the current system demonstrates efficient NIR-II fluorescence imaging and phototherapy, further efforts are required to enhance the excitation efficiency at longer wavelengths. Future work will focus on extending this copolymer strategy toward NIR–II excitation-responsive phototheranostic systems to achieve deeper tissue penetration and improved therapeutic performance. In addition, this design principle may potentially be extended to conventional rigid organic chromophores that suffer from aggregation-caused quenching by introducing controlled flexibility to improve their performance in aggregated states.

Moreover, although the observed tumor accumulation is likely dominated by passive enhanced permeability and retention (EPR) effects, further optimization of nanoparticle surface chemistry may enable active targeting strategies to improve the delivery specificity and therapeutic efficacy. Finally, comprehensive long-term biosafety and pharmacokinetic evaluations are necessary to support potential translational applications.

## 3 Conclusions

In conclusion, we present a modular copolymer strategy that effectively decouples NIR-II fluorescence, ROS generation, and photothermal conversion by integrating flexible and hydrogen-bond-reinforced rigid segments. This design addresses the long-standing trade-off between molecular rigidity and ACQ, enabling the retention of optical and therapeutic functions in nanoparticle states. Systematic variation of the segment ratio reveals that increasing the rigid-component content enhances NIR-II fluorescence and ROS generation while only slightly affecting photothermal efficiency, demonstrating that copolymer composition provides a programmable handle to balance multiple photophysical processes. Notably, the optimized F2R8 nanoparticles exhibit strong resistance to aggregation-induced quenching, maintaining high NIR-II emission and robust ROS production in the aggregated state. These features translate into effective *in vivo* performance, where F2R8 enables high-contrast NIR-II fluorescence imaging and imaging-guided synergistic phototherapy. Overall, this work establishes a generalizable strategy for designing quenching-resistant organic phototheranostic agents and highlights segment-level copolymer engineering as a powerful approach for tuning complex optical functions.

## 4 Experimental Section

### 4.1 Methods and Materials

The detailed characterization of the copolymers, including ^1^H NMR, elemental analysis, molecular weight, and polydispersity index (PDI), can be found in our previous work (https://pubs.acs.org/doi/10.1021/jacsau.5c00003). Absorption spectra of copolymers and their NPs were measured by an absorbance spectrometer (Shimadzu 1700). Fluorescence spectra of copolymers and NPs were measured by a spectrofluorometer (Edinburgh FLS980). The size distribution and ζ-potential of NPs were tested by a dynamic light scattering particle size analyzer (Malvem Zetasizer Nano ZS). Photothermal images and the temperatures of the solution samples were recorded by a thermal imaging camera (Fluke Ti400). Confocal images were collected using a laser confocal scanning microscope (Leica SPE).

### 4.2 Preparation of NPs

The stock solution of copolymers (1 mg/mL) is prepared by dissolving copolymers into THF. Pluronic F127 in THF (4 mL, 5 mg/mL) is then mixed with polymer stock solution (2 mL) by sonication. The mixture is rapidly dropped into deionized water (54 mL) under stirring. The mixture is stirred overnight to remove the THF solvent completely. Then, copolymer NP dispersion is ultrafiltered with centrifugal filter units (Millipore, size of 100 kDa) to obtain a concentrated NP solution. Finally, the concentrated copolymer NP dispersion is stored at 4 °C in the dark for long-term usage.

### 4.3 Determination of Fluorescence Quantum Yields

The photoluminescence quantum yield (PLQY) of copolymers and copolymer NPs are determined using IR-26 as a reference, with a PLQY value of 0.5% in 1,2-dichloroethane (DCE). IR-26 is dissolved in DCE to prepare five samples with different optical densities (ODs) at 808 nm (∼0.02, ∼0.04, ∼0.06, ∼0.08, and ∼0.1). Subsequently, the NIR-II fluorescence spectra are collected by the Edinburgh FLS980 and both the obtained spectra are integrated within the wavelength range of 900–1500 nm. The emission spectra of copolymers and their NP dispersions are obtained and integrated by the same method. Finally, the integrated fluorescence intensity is plotted against OD at 808 nm. The PLQY is calculated by

$$QY_{sample}=QY_{ref}\cdot \frac{slope_{sample}}{slope_{ref}}\cdot {\left(\frac{n_{sample}}{n_{ref}}\right)}^{2}$$

where *QY*_*sample*_ is the PLQY of copolymers/copolymer NPs with a wavelength of 900–1500 nm, *QY*_*ref*_ is the PLQY of IR-26, *slope*_*sample*_ is the slope obtained by linear fitting of the integrated fluorescence intensity of copolymers/copolymer NPs within the 900–1500 nm wavelength range against the OD at 808 nm, *slope*_*ref*_ is the slope obtained by linear fitting of the integrated fluorescence intensity of IR26 with a wavelength of 900–1500 nm against the OD at 808 nm, *n*_*sample*_ is the refractive indices of water, and *n*_*ref*_ is the refractive indices of DCE.

### 4.4 Determination of Quantum Yield of Singlet Oxygen Generation of NPs

The 1,3-diphenylisobenzofuran (DPBF) probe is used to characterize the singlet oxygen generation. Three milliliters of DI water containing ICG/NPs and DPBF probe is excited by 808 nm (330 mW/cm^2^) for 50 s with an interval of 10 s. The initial absorption of the DPBF probe in the testing solution is set as 1.0 at 415 nm, while the final optical density of molecules in the testing system is set as 0.180 at 808 nm. The experiments were conducted in the dark.

### 4.5 Detection of Intracellular ROS Generation

4T1 cells are seeded onto confocal dishes and incubated for a period of 24 h. The culture medium is then replaced with F2R8 NPs (60 μg/mL) and then incubated for another 4 h. Subsequently, the medium is discarded and replaced by DCFH-DA (10 μM) and Hoechst 33,342 (5 μM), followed by an additional incubation of 20 min. The laser-treated groups’ cells are excited using an 808 nm laser (1 W/cm^2^) for 5 min, after which the cells are washed three times by using PBS. Results are visualized using a laser confocal scanning microscope.

### 4.6 *In Vitro* Cytotoxicity Studies

For the cell counting kit 8 (CCK8) assay, 4T1 cells are seeded in 96-well plates at a density of 5000 cells each well. After incubating for 24 h, the culture medium is replaced with copolymer NPs dispersed in DMEM at various concentrations (100 μL; 0, 12, 24, 36, 48, 60 μg/mL). Following a subsequent incubation period of 4 h with the NPs, the laser-treated group is exposed to irradiation from an 808 nm laser (1 W/cm^2^) for 5 min. After another 24 h, the NP-containing medium is substituted with 10 μL of CCK-8 solution diluted in FBS-free DMEM (90 μL). The resulting mixture is then incubated for 1 h before measuring its OD at 450 nm using a microplate reader. Additionally, live/dead cell staining experiments are also conducted to validate the CCK8 results.

### 4.7 Tumor Model

The Balb/c mice utilized in the experiment are purchased from GemPharmatech Co., Ltd. All mice used are female, aged 5 weeks and weighing between 16 and 20 g. Subcutaneous injection of 50 μL 4T1 cell suspension (2 × 10^6^ cells/mL) is administered into the right leg of each mouse. Subsequent experimental procedures are conducted once the tumor volume reaches to 60 mm^3^. The ethics committee of Beijing Institute of Technology approved all mouse experiments (BIT-EC-SCXK (Jing) 2019–0010-M-056).

### 4.8 *In Vivo* NIR-II FLI

Mice with tumors are randomly selected for *in vivo* FLI. Following isoflurane gas anesthesia, the NIR-II small animal FLI system is used to detect the fluorescence signal of the tumor, representing the 0 h FLI result. Subsequently, F2R8 NPs (100 μL, 0.5 mg/mL) (*n* = 3) are intravenously injected into the mice and the fluorescence signals in mice are recorded at 3, 6, 12, 24, 36, 48 and 72 h, respectively. The experimental results are analyzed using ImageJ software. The highest time point for NP aggregation at the tumor site is determined at 36 h post-injection. For *in vitro* NIR-II imaging, the organs of mice treated with NPs were euthanized after 36 h, followed by immediate NIR-II imaging. The relative fluorescence intensity of different organs at various time point is detected by NirVivo-Pro device from BeiJing RayLight Technology Co., Ltd.

### 4.9 Histological Analysis

Organs including the heart, liver, spleen, lung, kidney, and tumor were extracted and fixed in a 4% paraformaldehyde solution for 24 h. All organs underwent hematoxylin–eosin (H&E) staining analysis for biosafety assessment purposes (*n* = 5). Besides, H&E staining along with terminal deoxynucleotidyl transfer-mediated dUTP Nick-end labeling assay staining is performed on tumors to evaluate treatment efficacy. The tumor of each group was subjected to H&E and TUNEL staining on the day 1 post-treatment.

### 4.10 Hematological Assay and Blood Biochemistry Assay

After 14 days of treatment, the blood of mice from different groups (PBS, PBS + L, F2R8 NPs, and F2R8 NPs + L) was collected. Whole blood is utilized for hematological analysis, while the serum is obtained through centrifugation of whole blood and used for blood biochemistry analysis (*n* = 5).

## Supplementary Material



## Notes

Theoretical calculations including binding energy, characterization of NP concentration, determination of relative PLQY, measurement of ROS generation, assessment of excited-state of dynamic behavior, calculation of photothermal properties, study of *in vivo* photothermal therapy, ACQ properties of organic small molecules, NP size and morphology analysis by DLS and TEM, NP stability studies, PL lifetimes of NPs, confocal fluorescence images of intracellular ROS generation, *in vivo* imaging studies, and histological analysis of major organs (PDF)
