# Supplementary material for: Decoupling Optical Functions via Ratio-Tunable Conjugated Copolymers with Hydrogen-Bond-Enforced Rigidity for Quenching-Resistant NIR-II Phototheranostics
Source: ACS Nano. 2026 Jul 13;20(29):20673–87. doi: 10.1021/acsnano.6c05195 (PMC13421988; doi:10.1021/acsnano.6c05195)
Supplement: Supplementary file 1 [file nn6c05195_si_001.pdf]

Decoupling Optical Functions via Ratio-Tunable Conjugated Copolymers with Hydrogen-Bond-Enforced Rigidity for Quenching-Resistant NIR-II Phototheranostics

Weilong Chen,†‡ Chuang Zhang,⁑ Guan-Lin Wu,₴ Ka-Wai Lee,†‡ Zhiqiang Guan,†‡ Yujuan Li,※ Bo-De Chen,₴ Chao Zhao,‡ Yung-Kang Peng,‡ Jinfeng Zhang,⁑ Chu-Chen Chueh,₴* Yingpeng Wan†‡**

†Center of Super-Diamond and Advanced Films (COSDAF), City University of Hong Kong

83 Tat Chee Avenue, Kowloon, Hong Kong SAR 999077, P. R. China

E-mail: ypwan3@cityu.edu.hk

‡Department of Chemistry, City University of Hong Kong

83 Tat Chee Avenue, Kowloon, Hong Kong SAR 999077, P. R. China

⁑Key Laboratory of Molecular Medicine and Biotherapy

School of Life Science, Beijing Institute of Technology

Beijing 100081, P. R. China

E-mail: jfzhang@bit.edu.cn

₴Department of Chemical Engineering, National Taiwan University, Taipei 10617, Taiwan

E-mail: cchueh@ntu.edu.tw

※School of Life Science, Beijing Institute of Technology, Haidian, Beijing, 100081, China

Table of Content

1. **Theoretical calculation** 3
2. **Determination of NPs concentration** 3
3. **Determination of relative PLQY** 3
4. **Assessment of reactive oxygen species generation** 3
5. **Excited-state dynamic behavior** 4
6. **Photothermal properties** 5
7. ***In vivo* photothermal therapy** 5
8. **Statistical analysis** 5
9. **Supplementary figures and tables** 6
10. **Reference** 25

1. Theoretical calculation

All theoretical calculations are performed on the Gaussian 16_A03 package.[1] The geometries of the ground (S_0) and singlet/triplet excited states (S_1/T_1) of the two segments are optimized using the M06-2X functional as well as the 6-311G(d) basis set. Transition dipole moments of two segments are obtained from the optimization of the singlet excited state. Relative energies of the specific dihedral angle rotation of flexible and rigid segments are scanned from the lowest energy point. Root-mean-square displacement (RMSD) values between S_0 and S_1 are calculated and visualized with the Visual Molecular Dynamic program (VMD1.9.3).[2]

According to the correlation analysis between atoms-in-molecules descriptors, binding energies, and energy components of symmetry-adapted perturbation theory, [3] neutral hydrogen bonding (HB) complexes are divided into "very weak" HBs whose BE magnitude is below 2.5 kcal/mol and "weak-to-medium" HBs, for which BE magnitude is larger than 2.5 kcal/mol but lower than 15.0 kcal/mol. On the other hand, charged HBs are classified as "medium" HBs with a BE magnitude between 11.0 and 15.0 kcal/mol and "strong" HBs with a BE magnitude greater than 15.0 kcal/mol. BE can be calculated by:

$$BE = -223.08 * \rho(BCP) + 0.7423 \quad (1)$$

where ρ(BCP) is the density of all electrons form specific hydrogen bonding.

2. Determination of NPs concentration

Concentration of NPs is measured by the standard curve of copolymers. Standard curves of copolymers in THF with varied concentrations are first obtained. Then, the optical densities of the absorption peak are plotted against solution concentration to get **Equation 2**. After that, NPs dispersion is dissolved back in THF and the corresponding optical density of the absorption peak is substituted into equation 1 to calculate the NPs concentration:

$$y = C + \varepsilon x \quad (2)$$

where y is optical density, x is concentration, C and ε are constant and extinction coefficient, respectively.

3. Determination of relative PLQY

Given that the absorption wavelength ranges differ among the molecules, applying identical excitation frequencies across all systems to evaluate anti-quenching behavior would not provide a reasonable comparison. To ensure methodological consistency, we therefore employed relative PLQY as the comparative parameter. In each experiment, the absorbance of both the single molecules and their NPs at a specified irradiation wavelength was carefully controlled to fixed values (approximately 0.02, 0.04, 0.06, 0.08, and 0.10). Relative PLQY was subsequently determined by integrating the fluorescence intensity at these absorbance levels and performing linear regression analysis. Finally, normalization of the relative PLQY values between the single molecules and their NPs yielded the results presented in **Figure 4D**.

4. Assessment of reactive oxygen species generation

Method of singlet oxygen detection of copolymers in THF (Figure S15): 1,3-Diphenylisobenzofuran (DPBF) probe is used to characterize the singlet oxygen generation. 3 mL THF solutions containing copolymers and DPBF probe are excited by 750 nm (300 mW/cm^2) for 60 seconds with an interval of 10 seconds. The initial absorption of the DPBF probe in the testing solution is set as 1.0 at 415 nm, while the final optical density of molecules in the testing system is set as 0.150 at 750 nm. The experiments are conducted in the dark.

Method of superoxide radicals detection of copolymers in THF (Figure S16): Dihydrorhodamine 123 (DHR 123) probe is utilized to detect the superoxide radicals. In 3 mL THF solution, 30 μL DHR 123 (stock concentration: 1mM) is added. The copolymer amount is adjusted to make the final optical density of the solution at 750 nm is 0.170. Both control and experimental group are exposed to 750 nm laser (300 mW/cm^2) with an interval of 10 seconds. The fluorescence intensity of DHR 123 is recorded under 460 nm excitation.

Method of hydroxyl radicals detection of copolymers in THF (Figure S17): Hydroxyphenyl fluorescein (HPF) probe (stock concentration: 2.36 mM) is used to verify the generation of hydroxyl radicals. 12.5 μL HPF is introduced into 3mL copolymer solution to carry on the experiments. The final optical density of copolymer solution at 750 nm is 0.170. Both control and experimental group are exposed to 750 nm laser (300 mW/cm^2) with an interval of 10 seconds. The fluorescence intensity of DHR 123 is recorded under 460 nm excitation.

Method of measuring total ROS-generation of NPs in water (Figure S18): To test the total reactive oxygen species (ROS) generation of NPs, 2',7'-dichlorodihydrofluorescein diacetate (DCFH-DA, 1.2 mg) is first dissolved in ethanol (2.5 mL) and mixed with NaOH (0.01 M, 10 mL). After 30 minutes, the mixture is diluted with phosphate-buffered saline (PBS, 50 mL) to prepare the DCFH stock solution stored at -20 °C in the dark for long-term use. Polymeric NPs dispersion is added into DCFH stock solution to obtain the final optical density of 0.200 at 750 nm. Then the NPs solution is excited by a 750 nm laser (300 mW/cm^2) with an interval of 20 seconds. The emission spectra of oxidized 2',7'-dichlorofluorescein (DCF) are collected by the Horiba-Duetta (λ_{ex} = 460 nm, λ_{em} = 480-700 nm).

Method of measuring singlet oxygen generation of F2R8 NPs in water by EPR (Figure 3G): The photoexcited generation of singlet oxygen was evaluated using an EPR spectrometer. Briefly, 20 μL of 2,2,6,6-tetramethylpiperidine (TEMP) probe solution was added to 3 mL of F2R8 NPs solution (50 μg/mL) and thoroughly mixed. The resulting mixture was irradiated with an 808 nm laser at a power density of 0.5 W/cm^2 for 5 min, followed by immediate EPR measurement.

5. Excited-state dynamic behavior

Excited-state dynamic behaviors of copolymer NPs were characterized by femtosecond transient absorption. The NPs samples (50 μg/mL) are excited by a 700 nm pump laser irradiation. According to normalized kinetic curve of copolymer NPs at representative wavelength of 808 nm within GSB region. The decays are fitted by the following function:

$$I = Ae^{-t/\tau}. \quad (3)$$

where t is the time delay between pump and probe pulses, τ is lifetime of the decay process.

6. Photothermal properties

Copolymer NPs dispersion is diluted to various concentrations in a total volume of 1 mL. Subsequently, the temperature of each sample is recorded during excitation with an 808 nm laser (1 W/cm^2) for 5 minutes. The photothermal conversion efficiency (PCE) of copolymer NPs is determined using **Equation (4)**, as described previously.[4] The temperature change of copolymer NPs (1 mL) with an absorption intensity at 808 nm ≈ 0.60 is obtained under 808 nm laser irradiation (1 W/cm^2). Once a plateau in temperature increase is reached, the laser is deactivated and the corresponding cooling temperature is recorded at intervals of every 30 seconds until the temperature returns to room temperature.

$$\eta = \frac{hS\Delta T_{max} - Q_{Dis}}{I(1 - 10^{-A_{808}})} \quad (4)$$

where h represents the heat transfer coefficient, S represents the container area, ΔT_{max} indicates the temperature difference between peak flat-state temperature and room temperature, Q_{Dis} indicates the heat relevant to the light absorbance of deionized water, I indicates the excitation power density, and A_{808} indicates the absorption intensity of F2R8 NPs at 808 nm. hS can be obtained by **Equation (5)**.

$$\tau_s = \frac{m_D C_D}{hS} \quad (5)$$

where m_D represents the mass of water (1 g), and C_D represents the heat capacity of water (4.2 J/g). τ_s can be obtained by **Equation (6)**.

$$t = -\tau_s(ln\ \theta) \quad (6)$$

where θ is the ratio of ΔT to ΔT_{max}, ΔT is the temperature difference between the cooling temperature at 30 seconds intervals and room temperature. τ_ss of F8R2, F5R5, and F2R8 NPs are determined to be 350, 354, and 360 seconds.

7. *In vivo* photothermal therapy

The mice were randomly divided into PBS, PBS+L, F2R8 NPs, and F2R8 NPs+L groups (n=5 for each group). PBS and PBS+L groups were intravenously injected with 100 μL PBS. F2R8 NPs and F2R8 NPs+L groups were intravenously injected with 100 μL F2R8 NPs (dissolved in PBS, 1 mg/mL). After 36 hours of injection, the tumor site was irradiated with 808 nm laser (1 W/cm^2 for 5 min). The weight and tumor volume of the mice were recorded every two days after treatment. All mice were sacrificed on day 14, and the tumors were weighed and monitored very two days across all experimental groups using the formula: volume = (length * width^2)/2.

8. Statistical analysis

The optical spectra of polymers and NPs are normalized by Origin. Data shown are mean ± standard deviation. The sample size for each statistical analysis is listed in corresponding figure legends. One-way ANOVA with Tukey test is carried out across

groups using Origin. In all cases, significances are defined as $p \leq 0.05$.

9. Supplementary figures and tables

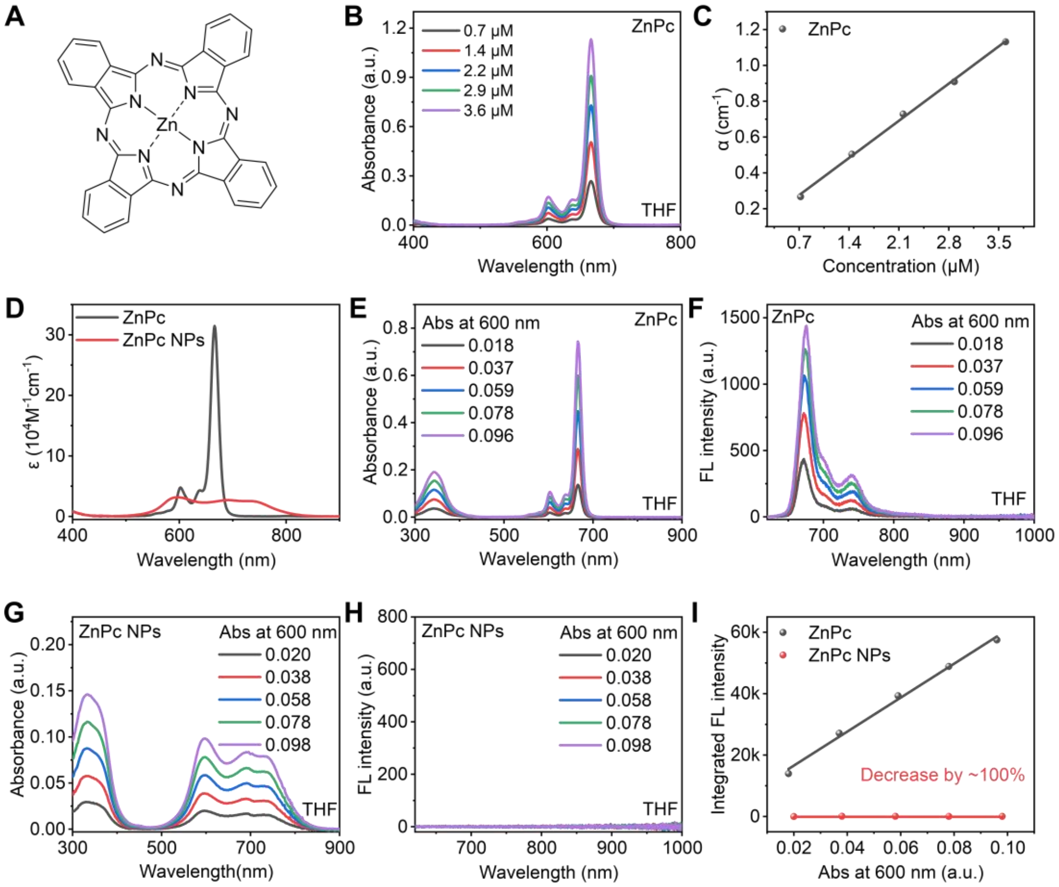

Figure S1. ACQ properties of organic small molecule ZnPc. (A) molecular structure of ZnPc. (B) Standard curve of ZnPc in THF. (C) Plot of optical densities of absorption maxima against concentrations to determine molar extinction coefficient. (D) UV-*vis*-NIR absorption spectra of ZnPc and ZnPc NPs. (E) Absorption spectra of ZnPc in THF with different OD values at 600 nm. (F) Fluorescence spectra (from 620-1000 nm) of ZnPc in THF corresponding to different OD at 600 nm. (G) Absorption spectra of ZnPc NPs in water with different OD values at 600 nm. (H) Fluorescence spectra (from 620-1000 nm) of ZnPc NPs in water corresponding to different OD at 600 nm. (I) Plots of integrated fluorescence intensities (620-1000 nm) of ZnPc and ZnPc NPs at five concentrations.

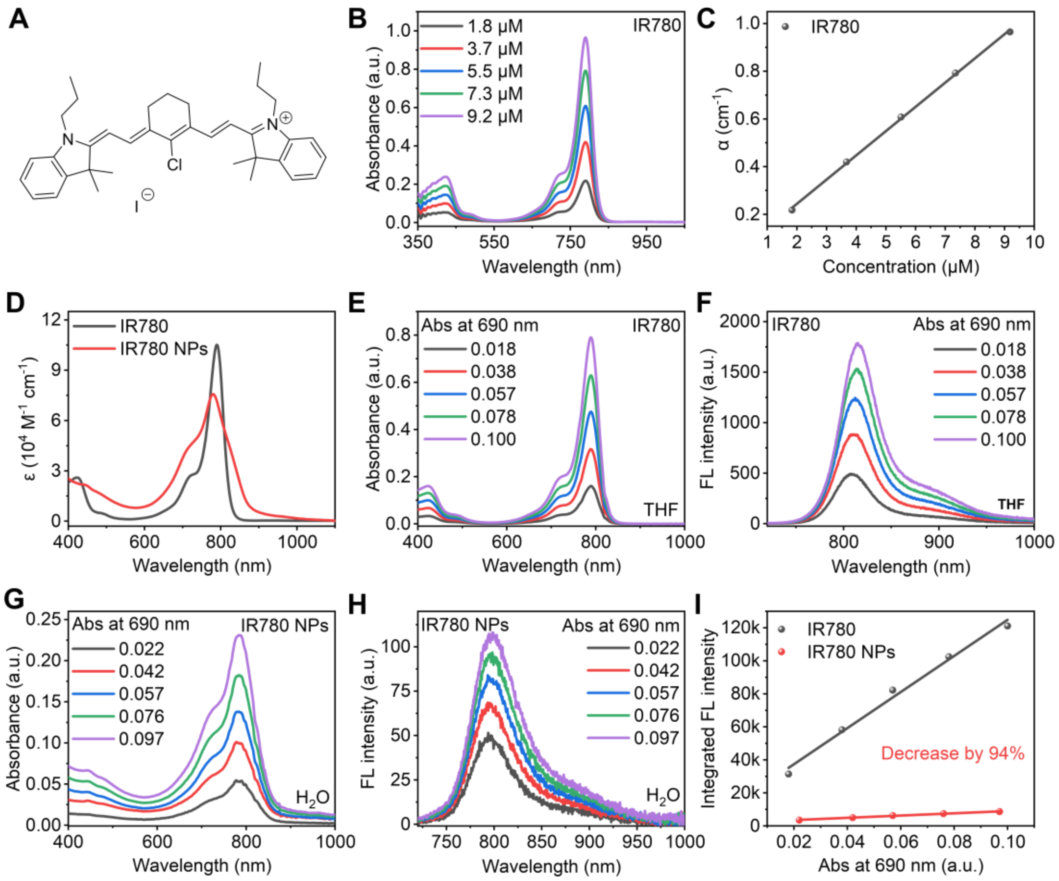

Figure S2. ACQ properties of organic small molecule IR780. (A) molecular structure of IR780. (B) Standard curve of IR780 in THF. (C) Plot of optical densities of absorption maxima against concentrations to determine molar extinction coefficient. (D) UV-*vis*-NIR absorption spectra of IR780 and IR780 NPs. (E) Absorption spectra of IR780 in THF with different OD values at 690 nm. (F) Fluorescence spectra (from 720-1000 nm) of IR780 in THF corresponding to different OD at 690 nm. (G) Absorption spectra of IR780 NPs in water with different OD values at 690 nm. (H) Fluorescence spectra (from 720-1000 nm) of IR780 NPs in water corresponding to different OD at 690 nm. (I) Plots of integrated fluorescence intensities (720-1000 nm) of IR780 and IR780 NPs at five concentrations.

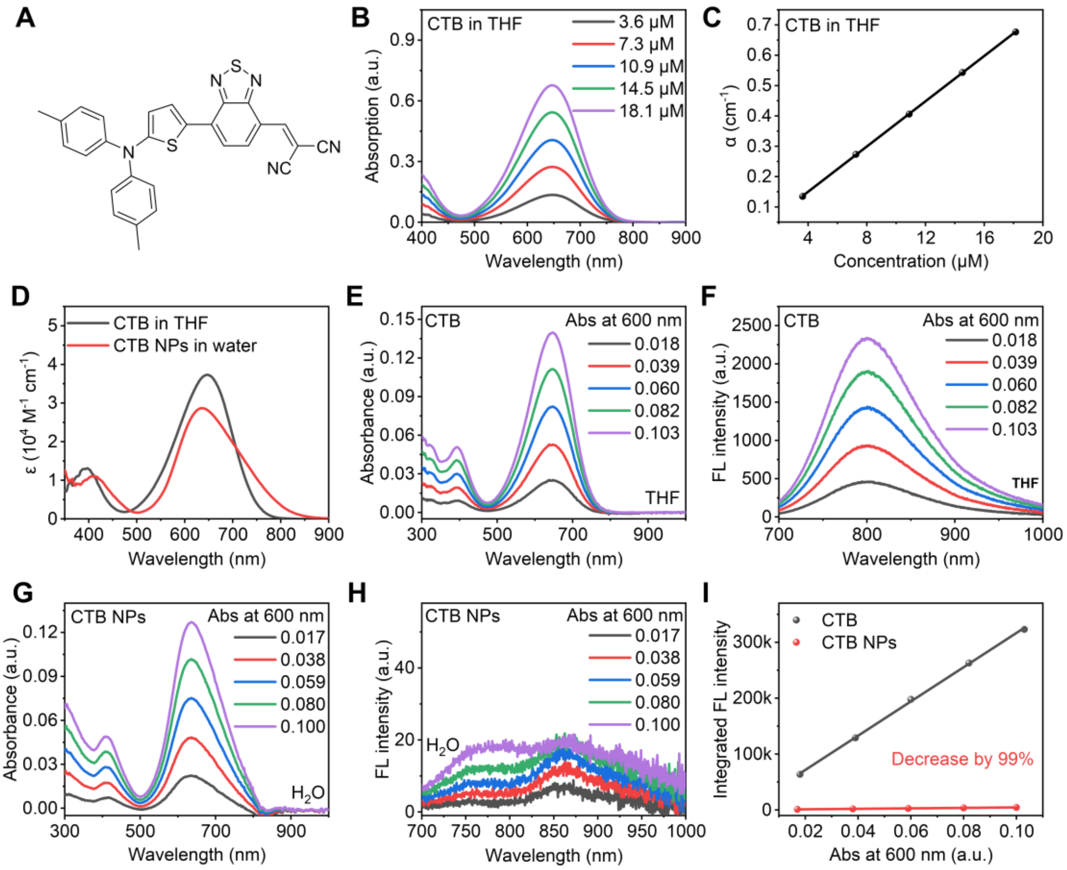

Figure S3. ACQ properties of organic small molecule CTB. (A) molecular structure of CTB. (B) Standard curve of CTB in THF. (C) Plot of optical densities of absorption maxima against concentrations to determine molar extinction coefficient. (D) UV-*vis*-NIR absorption spectra of CTB and CTB NPs. (E) Absorption spectra of CTB in THF with different OD values at 600 nm. (F) Fluorescence spectra (from 700-1000 nm) of CTB in THF corresponding to different OD at 600 nm. (G) Absorption spectra of CTB NPs in water with different OD values at 600 nm. (H) Fluorescence spectra (from 700-1000 nm) of CTB NPs in water corresponding to different OD at 600 nm. (I) Plots of integrated fluorescence intensities (700-1000 nm) of CTB and CTB NPs at five concentrations.

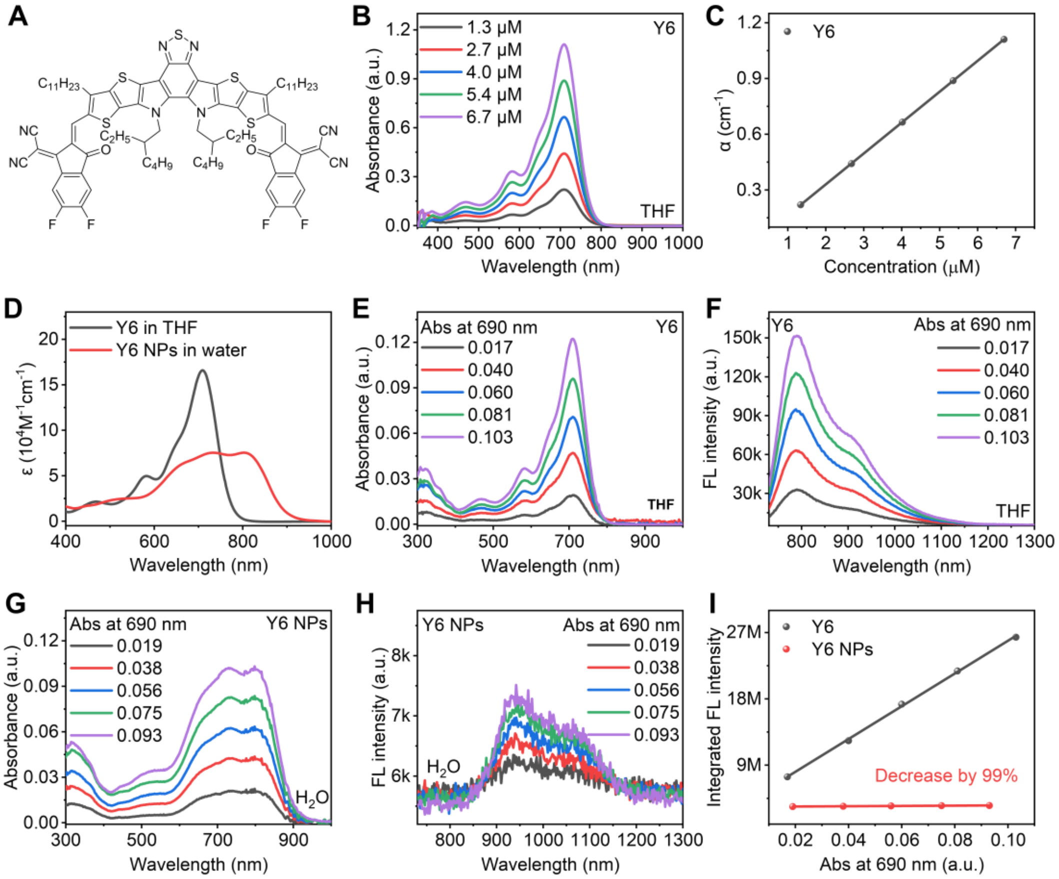

Figure S4. ACQ properties of organic small molecule Y6. (A) molecular structure of Y6. (B) Standard curve of Y6 in THF. (C) Plot of optical densities of absorption maxima against concentrations to determine molar extinction coefficient. (D) UV-*vis*-NIR absorption spectra of Y6 and Y6 NPs. (E) Absorption spectra of Y6 in THF with different OD values at 690 nm. (F) Fluorescence spectra (from 730-1000 nm) of Y6 in THF corresponding to different OD at 690 nm. (G) Absorption spectra of Y6 NPs in water with different OD values at 690 nm. (H) Fluorescence spectra (from 730-1000 nm) of Y6 NPs in water corresponding to different OD at 690 nm. (I) Plots of integrated fluorescence intensities (730-1000 nm) of Y6 and Y6 NPs at five concentrations.

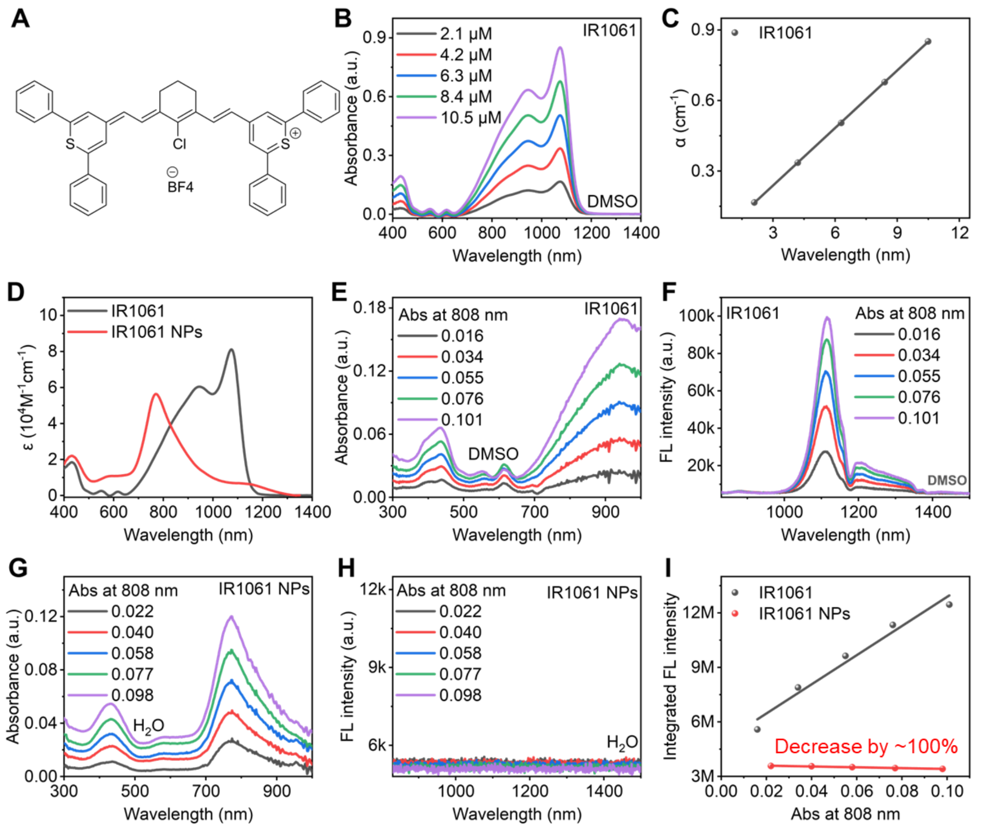

Figure S5. ACQ properties of organic small molecule IR1061. (A) molecular structure of IR1061. (B) Standard curve of IR1061 in DMSO. (C) Plot of optical densities of absorption maxima against concentrations to determine molar extinction coefficient. (D) UV-*vis*-NIR absorption spectra of IR1061 and IR1061 NPs. (E) Absorption spectra of IR1061 in DMSO with different OD values at 808 nm. (F) Fluorescence spectra (from 830-1500 nm) of IR1061 in DMSO corresponding to different OD at 808 nm. (G) Absorption spectra of IR1061 NPs in water with different OD values at 808 nm. (H) Fluorescence spectra (from 830-1500 nm) of IR1061 NPs in water corresponding to different OD at 808 nm. (I) Plots of integrated fluorescence intensities (830-1500 nm) of IR1061 and IR1061 NPs at five concentrations.

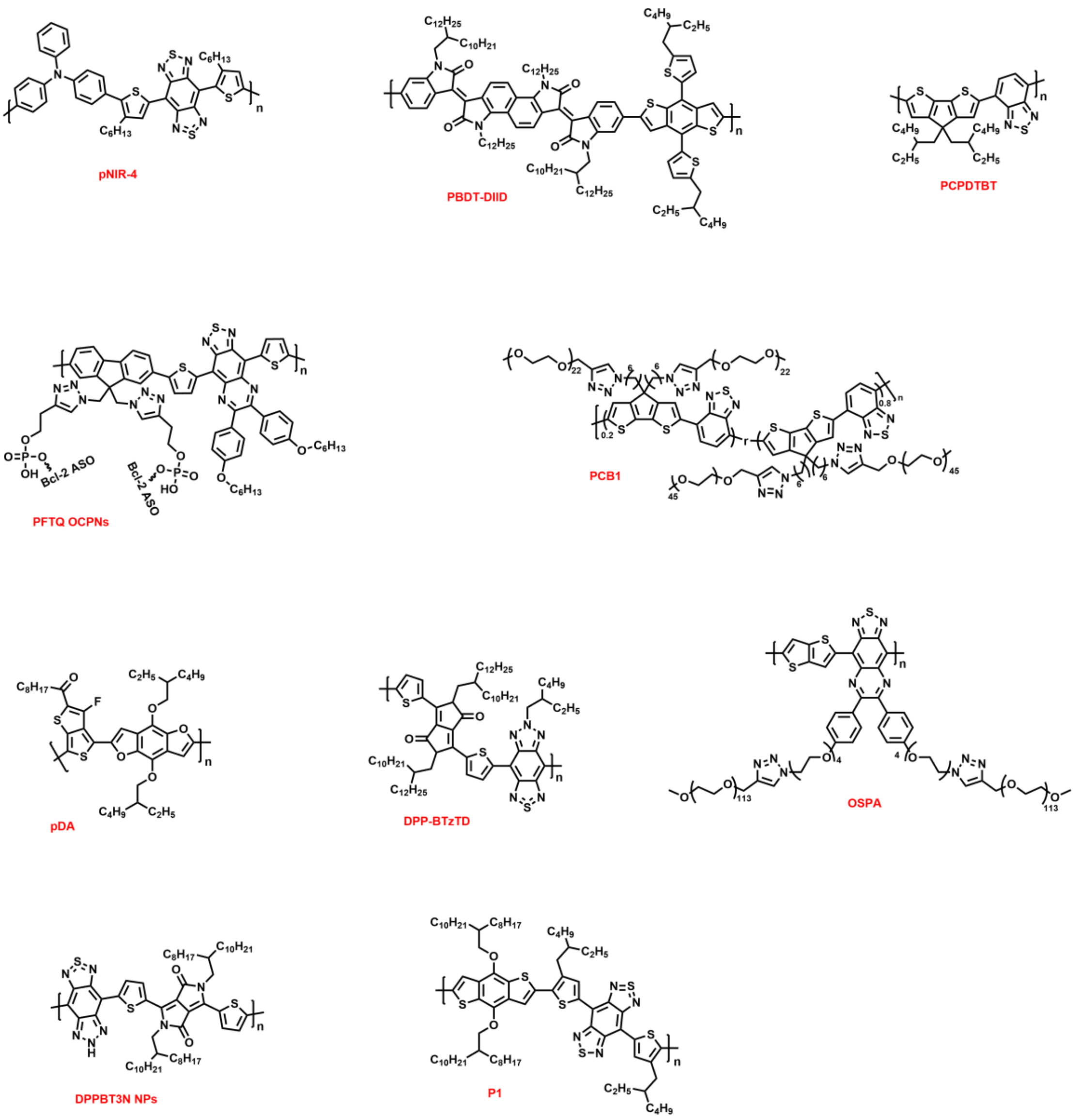

Figure S6. Representative conjugated polymers reported for phototheranostic applications.

Table S1. Summary of representative conjugated polymers reported for phototheranostic applications, compared with the copolymer developed in this work.

Polymers	λ_{Ex} (nm)	Φ_F (%)	ROS generation	PCE (%)	References
pNIR-4 NPs	709	2.24	-	-	*J. Am. Chem. Soc.* ***2020***, 142, 15146.
PBDT-DIID NPs	808	-	-	70.6	*Adv. Mater.* ***2022***, 34, 2200179.
PCPDTBT NPs	808	-	√	34.9	*Macromolecules* ***2022***, 56, 311.
PFTQ OCPNs	808	0.30	-	69.8	*Angew. Chem. -In. Ed.* ***2025***, 64, e202425654.
PCB1	808	-	√	42.8	*Angew. Chem. -In. Ed.* ***2018***, 57, 3995.
pDA-PEG	808	~1.7	-	-	*Nat. Commun.* ***2014***, 5, 4206.
DPPBT3N NPs	1064	-	-	45.2	*Angew. Chem. -In. Ed.* ***2025***, e202511084.
DPP-BTzTD NPs	1064	-	-	53	*Adv. Funct. Mater.* ***2020***, 30, 1909673.
OSPA	1064	-	-	30.53	*Biomaterials* ***2020***, 232, 119684.
P1 NPs	1064	-	-	30.1	*Adv. Mater.* ***2018***, 30, 1802591.
F2R8 NPs	808	1.68	√	41.8	*This work*

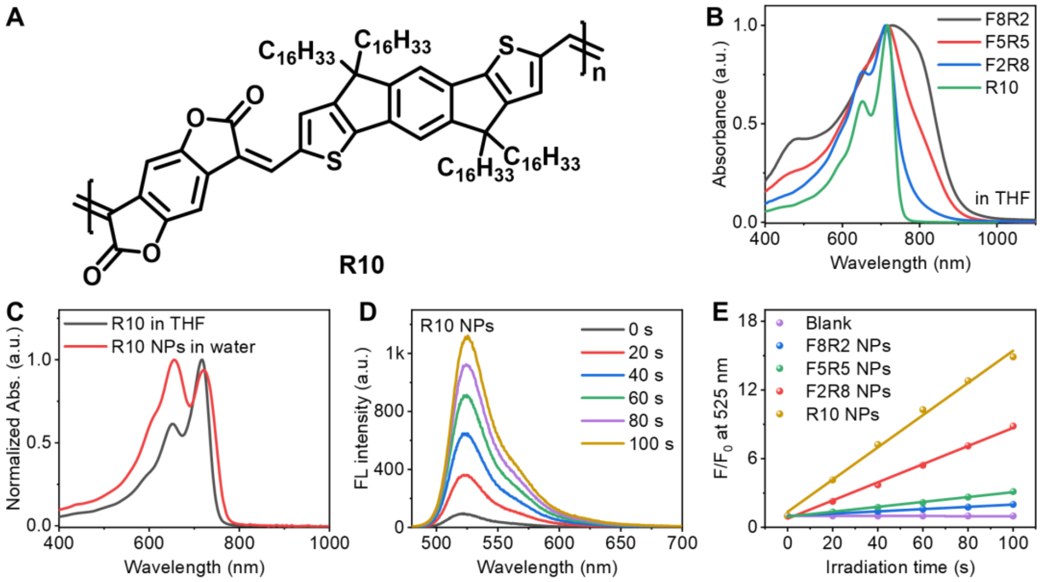

Figure S7. (A) Molecular structure of polymer R10. (B) Normalized UV-*vis*-NIR absorption spectra of F8R2, F5R5, F2R8, and R10 in THF. (C) Normalized absorbance

of R10 in THF and R10 NPs in water. (D) Time-dependent fluorescence intensity changes of DCFH with R10 NP aqueous solution upon 750 nm irradiation (power density: 300 mW/cm^2). (E) Linear fitting of relative DCFH fluorescence intensities of the four NPs under 750 nm excitation at different time points.

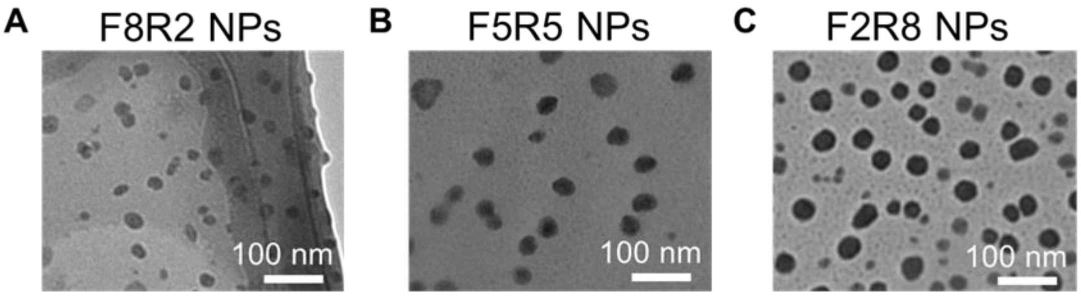

Figure S8. Morphology of prepared F8R2 NPs (A), F5R5 NPs (B), and F2R8 NPs (C) obtained by TEM.

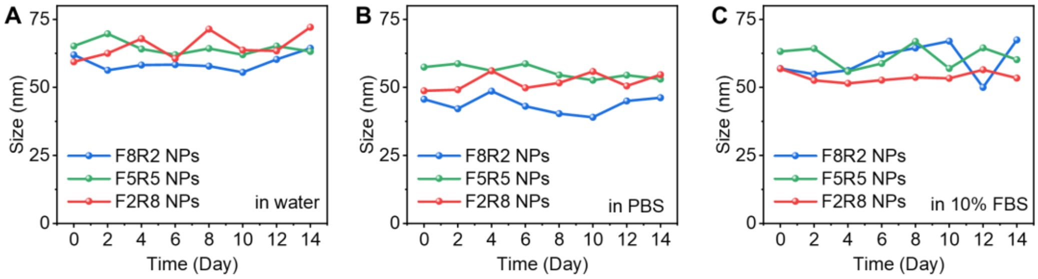

Figure S9. Size distributions of F8R2, F5R5, F2R8 NPs dispersed in DI water (A), phosphate-buffered saline (PBS) (B), and 10% fetal bovine serum (FBS) (C) over 14 days.

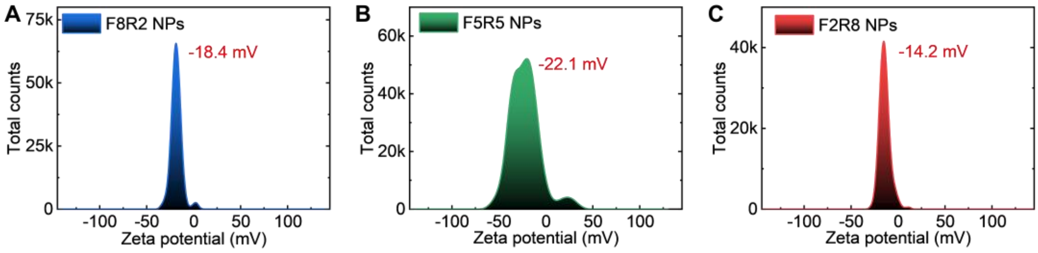

Figure S10. Zeta potential of prepared F8R2, F5R5, F2R8 NPs dispersed in DI water.

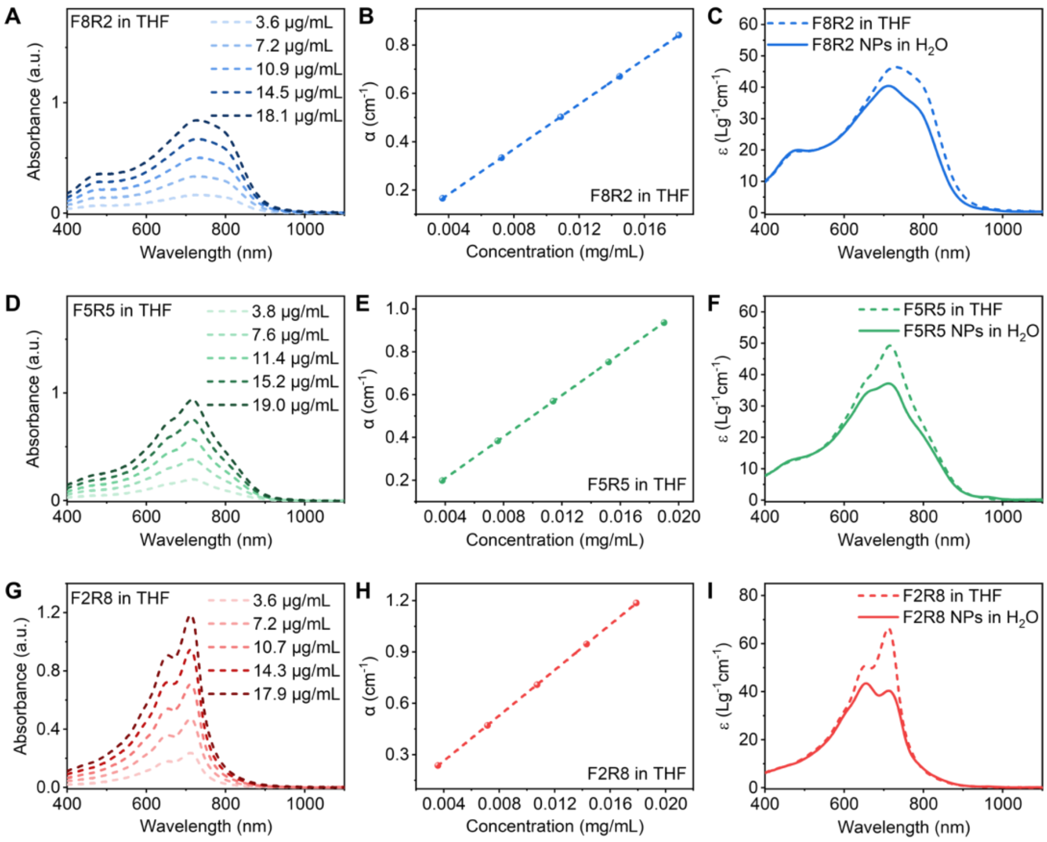

Figure S11. Measurement of absorbance for F8R2, F5R5, and F2R8 and their corresponding NPs. (A) Standard curve of F8R2 in THF. (B) Plot of optical density at the absorption maxima versus concentration for determining the molar extinction coefficient of F8R2. (C) Molar extinction coefficients of F8R2 and F8R2 NPs. (D) Standard curve of F5R5 in THF. (E) Plot of optical density at the absorption maxima versus concentration for determining the molar extinction coefficient of F5R5. (F) Molar extinction coefficients of F5R5 and F5R5 NPs. (G) Standard curve of F2R8 in THF. (H) Plot of optical density at the absorption maxima versus concentration for determining the molar extinction coefficient of F2R8. (I) Molar extinction coefficients of F2R8 and F2R8 NPs.

Table S2. Summary of photophysical properties of copolymers and corresponding NPs.

	λ_{Abs} (nm)	λ_{Em} (nm)	Φ_F (%)	ROS generation	PCE (%)
F8R2	729	-	0.55	-	-
F8R2 NPs	712	1010	0.44	1 time	42.9
F5R5	716	-	0.97	-	-
F5R5NPs	711	1012	0.65	2.2 times of F8R2 NPs	42.3
F2R8	711	-	2.80	-	-
F2R8 NPs	656	918	1.68	8.2 times of F8R2 NPs	41.4

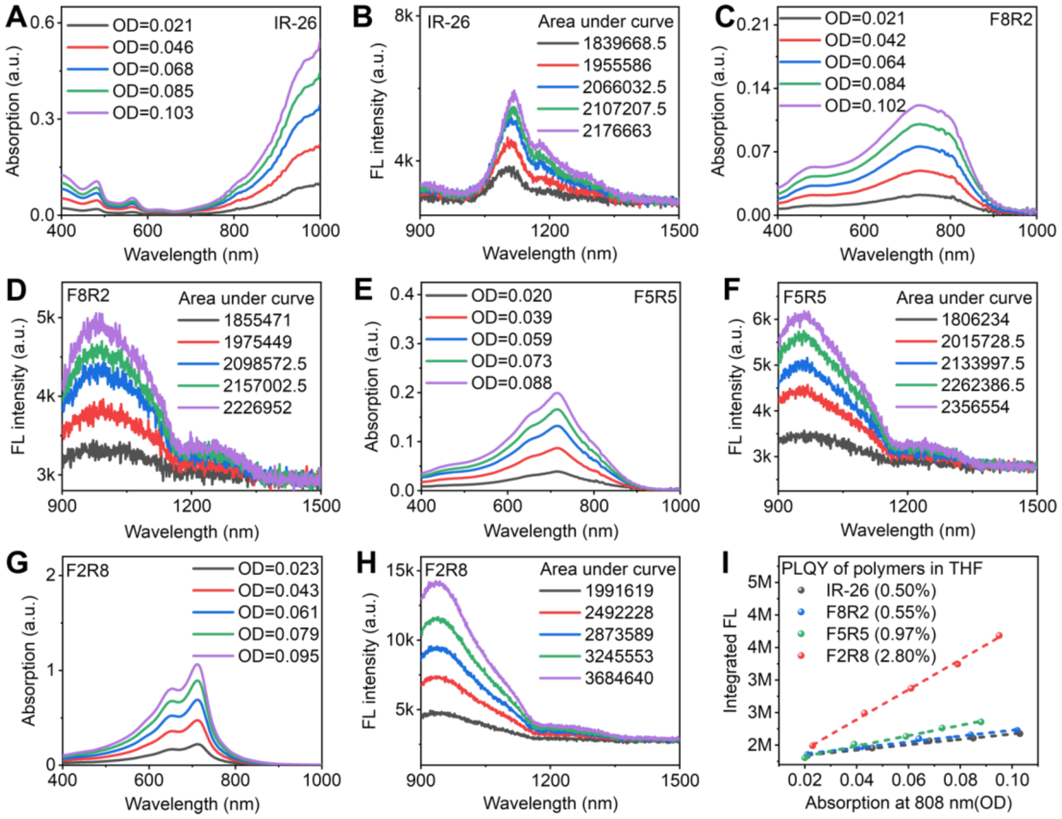

Figure S12. Absorption spectra of IR-26 (A), F8R2 (C), F5R5 (E), and F2R8 (G) with OD values at 808 nm. Fluorescence spectra of IR-26 (B), F8R2 (D), F5R5 (F), and F2R8 (H) with integrated NIR-II fluorescence intensities from 900-1500 nm. Excitation source: 808 nm laser. (I) Plots of integrated fluorescence intensities (900-1500 nm) of IR-26, F8R2, F5R5, and F2R8 in THF at five concentrations.

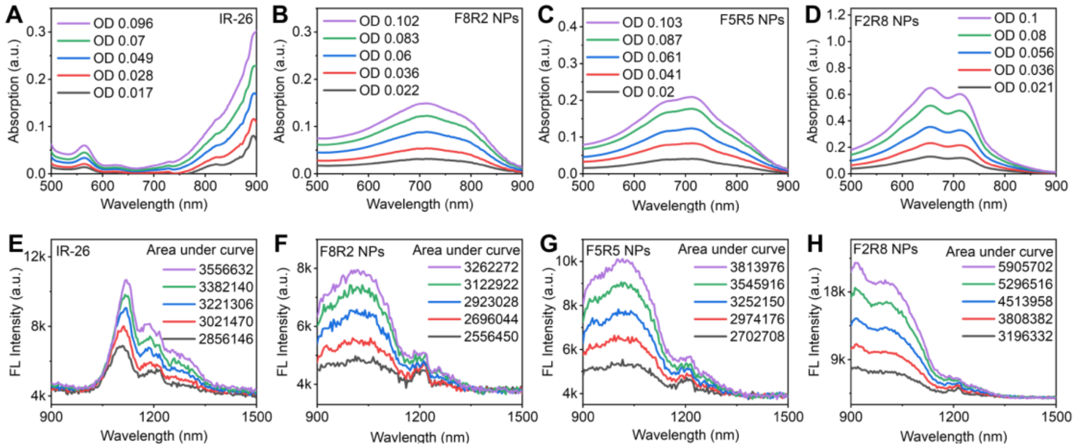

Figure S13. Absorption spectra of IR-26 (A), F8R2 NPs (B), F5R5 NPs (C), and F2R8 NPs (D) with OD values at 808 nm. Fluorescence spectra of IR-26 (E), F8R2 NPs (F), F5R5 NPs (G), and F2R8 NPs (H) with integrated NIR-II fluorescence intensities from 900-1500 nm. Excitation source: 808 nm laser.

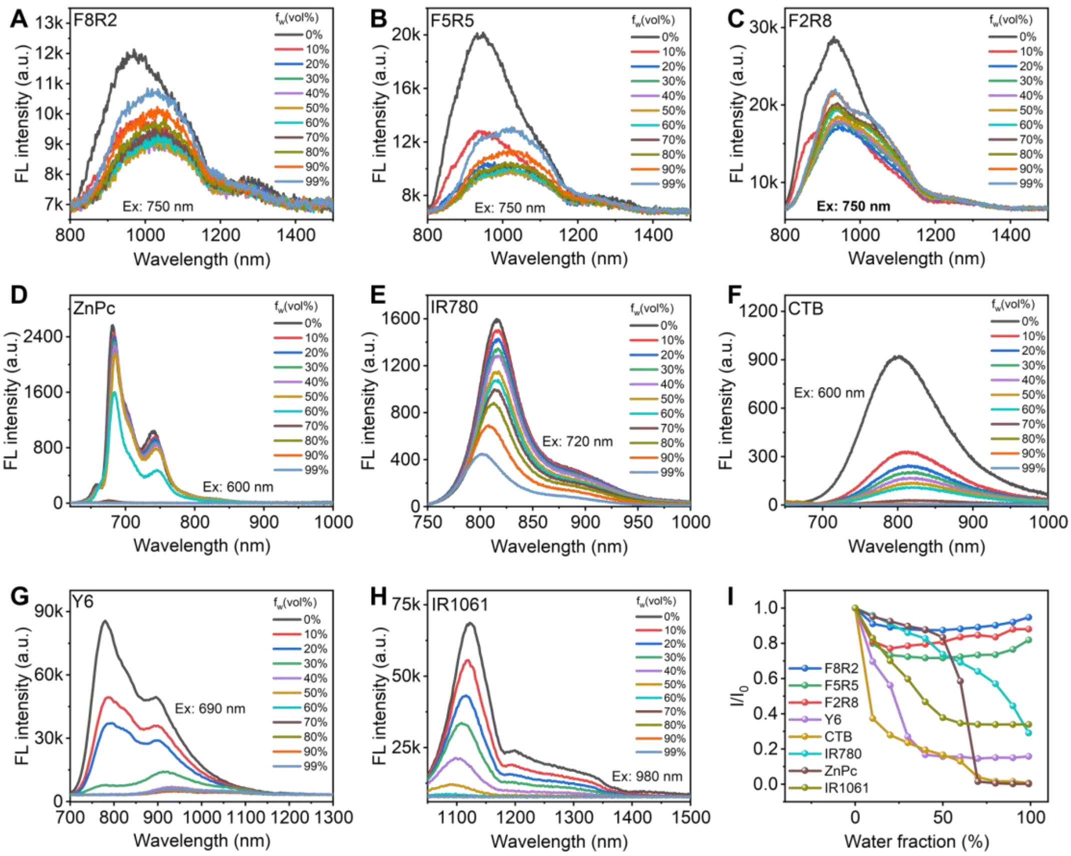

Figure S14. Photoluminescence spectra of obtained copolymers and commercial organic small molecules in a mixture of THF/DMSO (only for IR1061) and water with varying water fractions (f_w). (A) F8R2. (B) F5R5. (C) F2R8. (D) ZnPc. (E) IR780. (F) CTB. (G) Y6. (H) IR1061. (I) Plots of I/I_0 *vs.* f_w, where I_0s are the integration of polymers and organic small molecules in pure THF/DMSO solution.

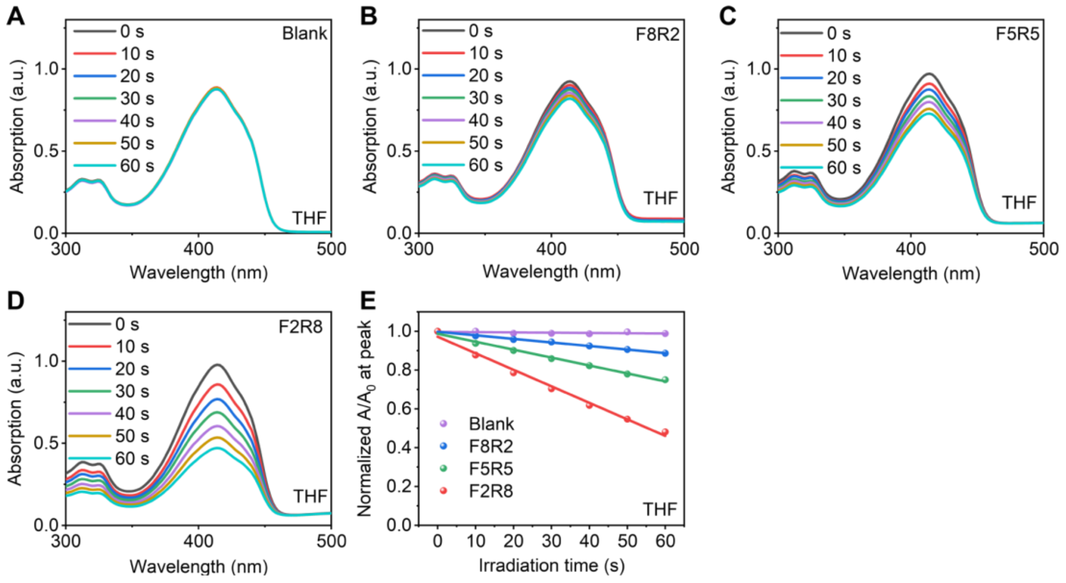

Figure S15. Singlet oxygen detection. Time-dependent absorption spectra of DPBF probe with DPBF only in THF (A), F8R2 in THF (B), F5R5 in THF (C), and F2R8 in THF (D) under 750 nm laser excitation (power density: 300 mW/cm^2). (E) Summary data of A-D.

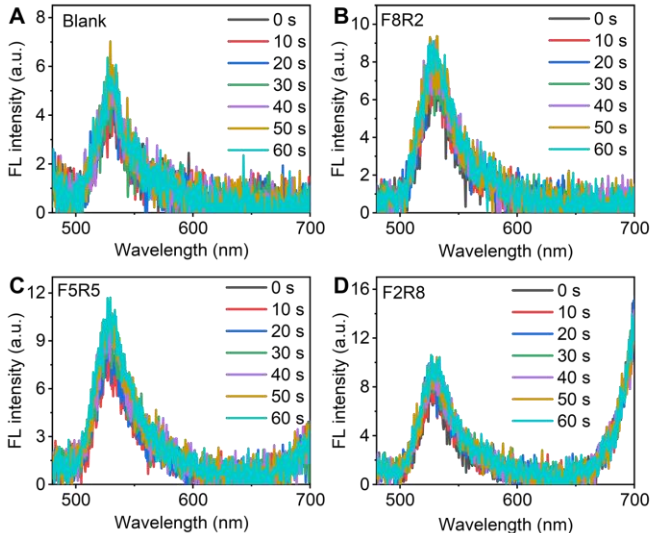

Figure S16. Superoxide radicals' detection of obtained copolymers. Time-dependent fluorescence spectra of DHR123 probe with DHR123 only in THF (A), F8R2 in THF (B), F5R5 in THF (C), and F2R8 in THF (D) under 750 nm laser excitation (power density: 300 mW/cm^2).

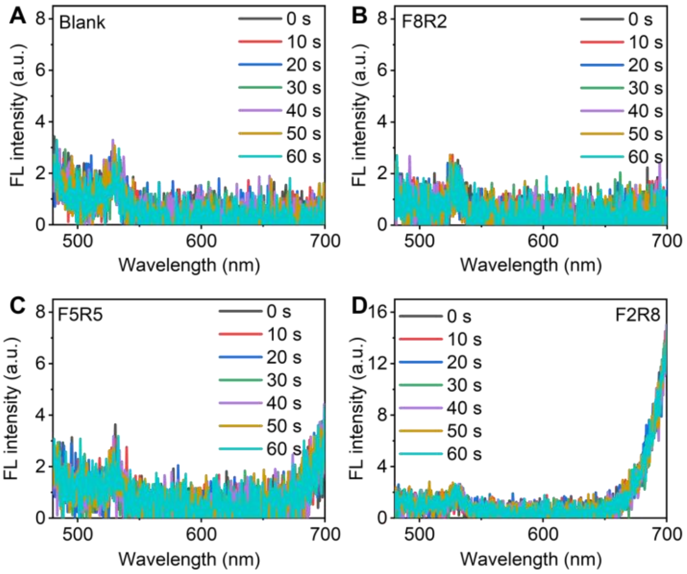

Figure S17. Hydroxyl radicals' detection of obtained copolymers. Time-dependent fluorescence spectra of HPF probe in THF (A), F8R2 in THF (B), F5R5 in THF (C), and F2R8 in THF (D) under 750 nm laser excitation (power density: 300 mW/cm^2).

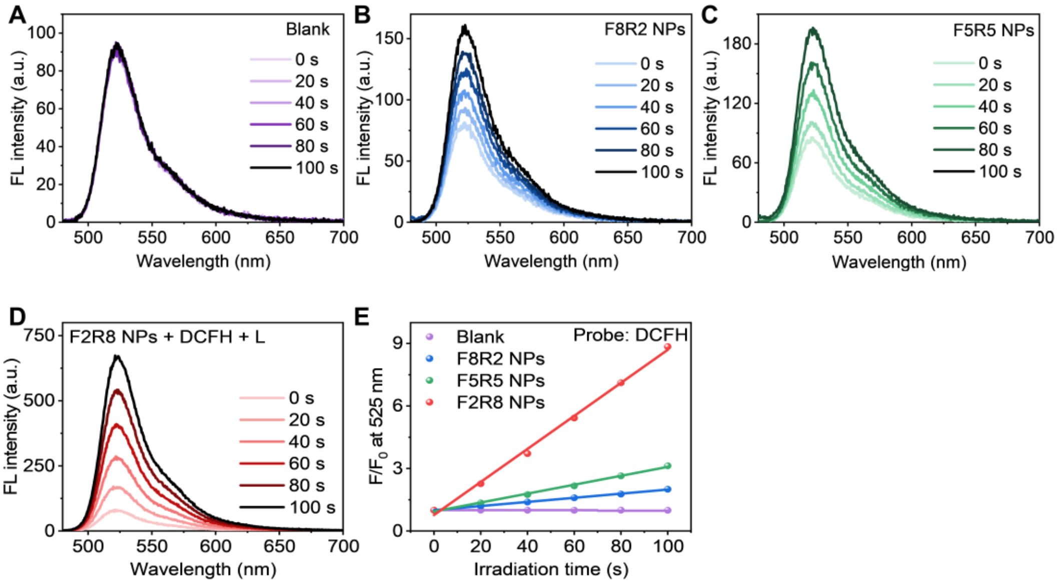

Figure S18. Time-dependent fluorescence intensity changes of DCFH with blank (A), F8R2 NP (B), F5R5 NP (C), and F2R8 NPs (D) aqueous solution upon 750 nm irradiation (power density: 300 mW/cm^2). (E) Summary data of A-D.

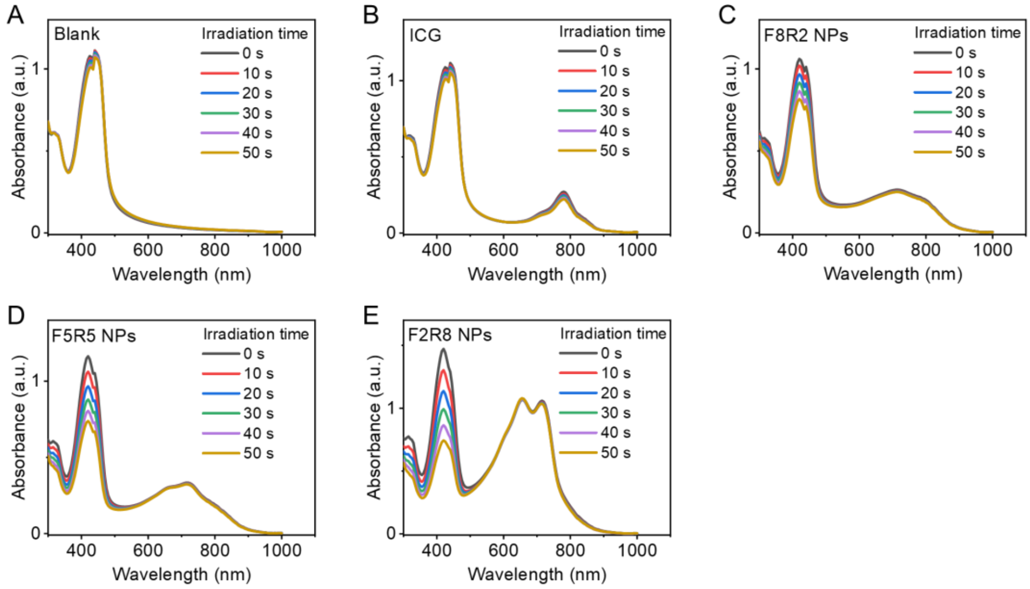

Figure S19. Quantitative singlet oxygen detection. Time-dependent absorption spectra of DPBF probe with DPBF only in water (A), ICG in water (B), F8R2 NPs in water (C), F5R5 NPs in water (D), and F2R8 NPs (E) under 808 nm laser excitation (power density: 330 mW/cm^2).

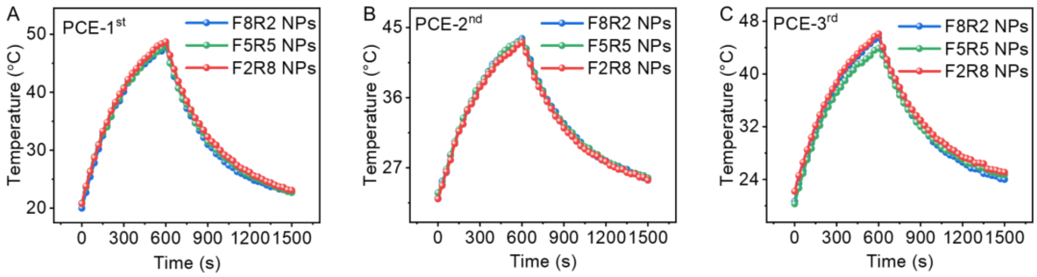

Figure S20. Heating and cooling curves of F8R2, F5R5, and F2R8 NPs with the similar absorbance at 808 nm under 808 nm laser irradiation (1W/cm^2) for the first (A), the second (B) and the third (C) time.

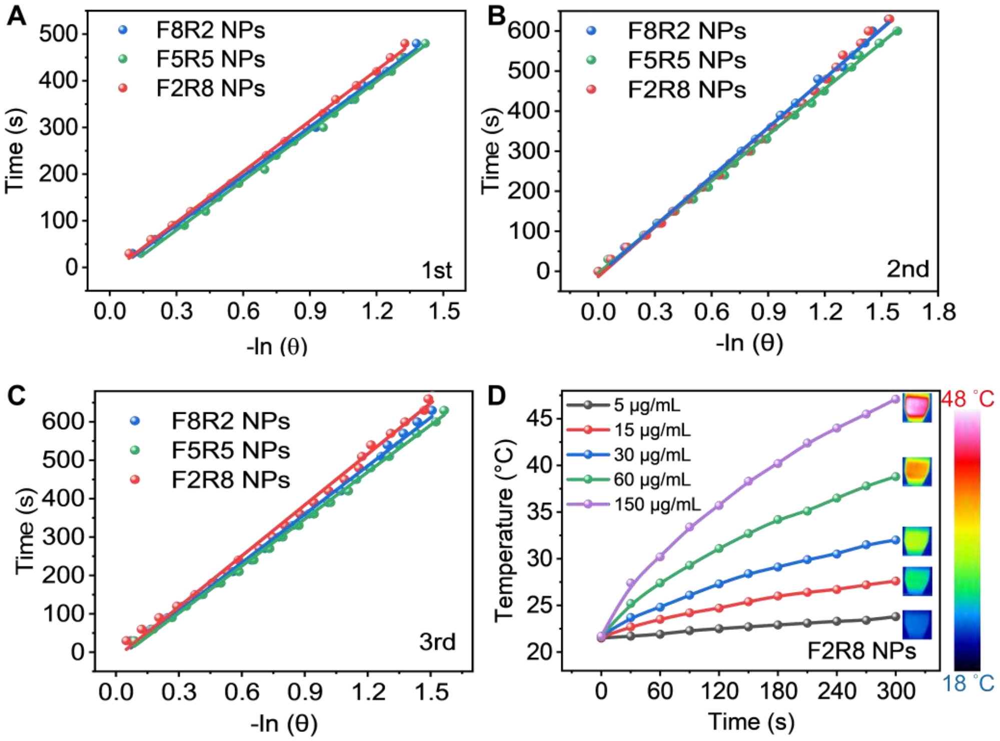

Figure S21. (A) Cooling time *vs* -ln(θ) plot of copolymer NPs for the first time. (B) Cooling time *vs* -ln(θ) plot of copolymer NPs for the second time. (C) Cooling time *vs* -ln(θ) plot of copolymer NPs for the third time. (D) Temperature-increasing curve of F2R8 NPs with varied concentrations after 5 min of laser irradiation with corresponding infrared thermographs.

Table S3. Summary of parameters determining the PCE of copolymer NPs for the second time.

PCE-1st	F8R2 NPs	F5R5 NPs	F2R8 NPs
Abs@808 nm	0.598	0.596	0.604
ΔT_{max} (°C)	27.8	27.7	27.9
τ_s (s)	350	354	361
Q_{dis} (W)	0.013	0.013	0.013
I (W/cm^2)	1	1	1
PCE (%)	42.9	42.3	41.4

Table S4. Summary of parameters determining the PCE of copolymer NPs for the second time.

PCE-2nd	F8R2 NPs	F5R5 NPs	F2R8 NPs
Abs@808 nm	0.325	0.323	0.312
ΔT_{max} (°C)	20.5	19.5	19.2
τ_s (s)	379	384	390
Q_{dis} (W)	0.009	0.009	0.009
I (W/cm^2)	1	1	1
PCE (%)	41.2	38.7	38.4

Table S5. Summary of parameters determining the PCE of copolymer NPs for the third time.

PCE-3rd	F8R2 NPs	F5R5 NPs	F2R8 NPs
Abs@808 nm	0.425	0.395	0.403
ΔT_{max} (°C)	24.8	23.6	23.9
τ_s (s)	395	408	412
Q_{dis} (W)	0.007	0.007	0.007
I (W/cm^2)	1	1	1
PCE (%)	41.0	39.5	39.1

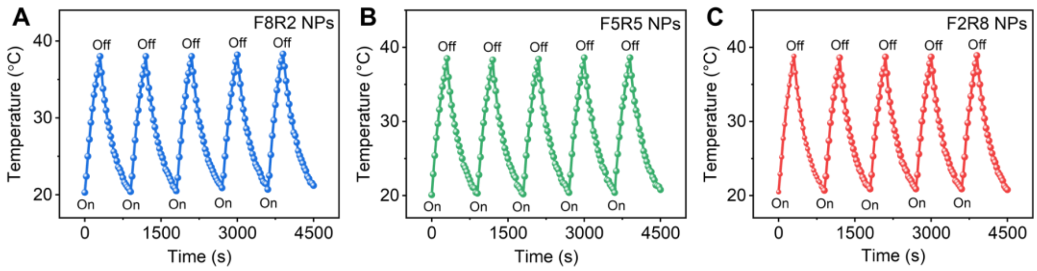

Figure S22. Photostability tests of F8R2 NPs (A), F5R5 NPs (B), and F2R8 NPs (C) for five cycles.

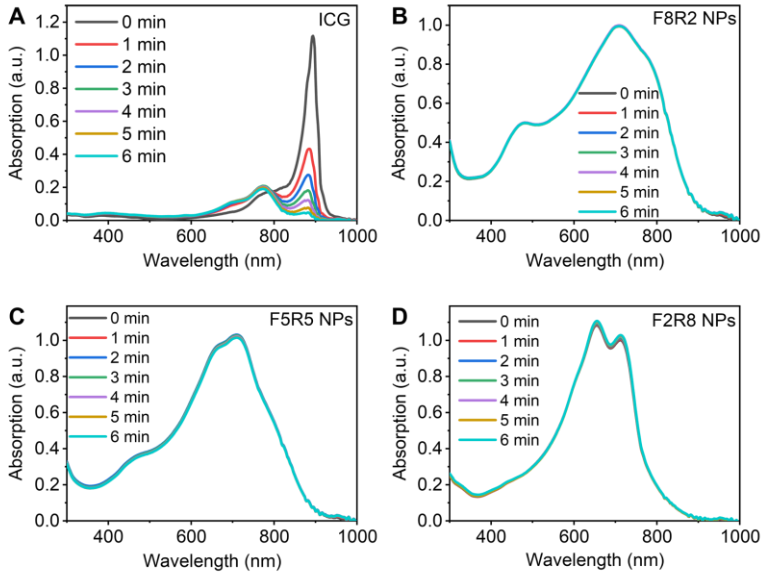

Figure S23. Absorption stability of ICG (A), F8R2 NPs (B), F5R5 NPs (C), and F2R8 NPs (D) in water upon continuous laser (808 nm, 500 mW/cm^2) irradiation for 6 min.

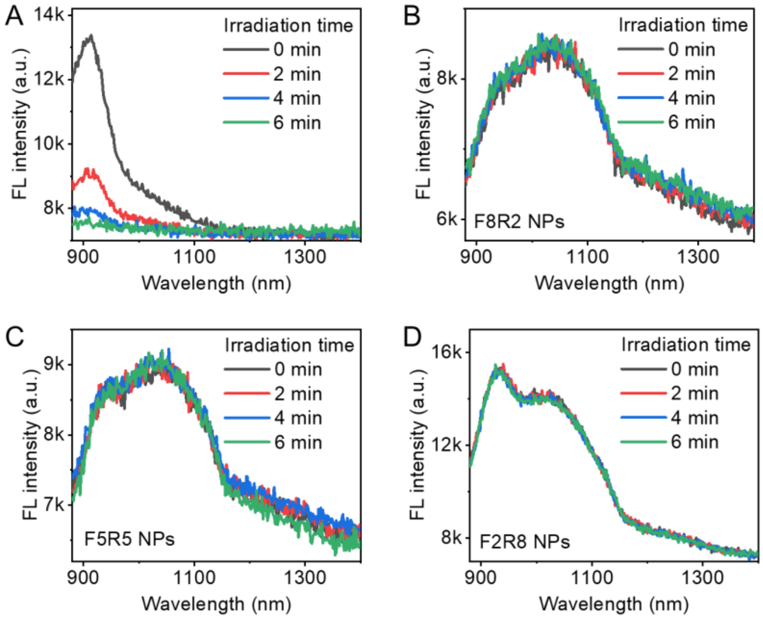

Figure S24. Emission stability of ICG (A), F8R2 NPs (B), F5R5 NPs (C), and F2R8 NPs (D) in water upon continuous laser (808 nm, 500 mW/cm^2) irradiation for 6 min. The absorbance at 808 nm of each sample is set as 0.2.

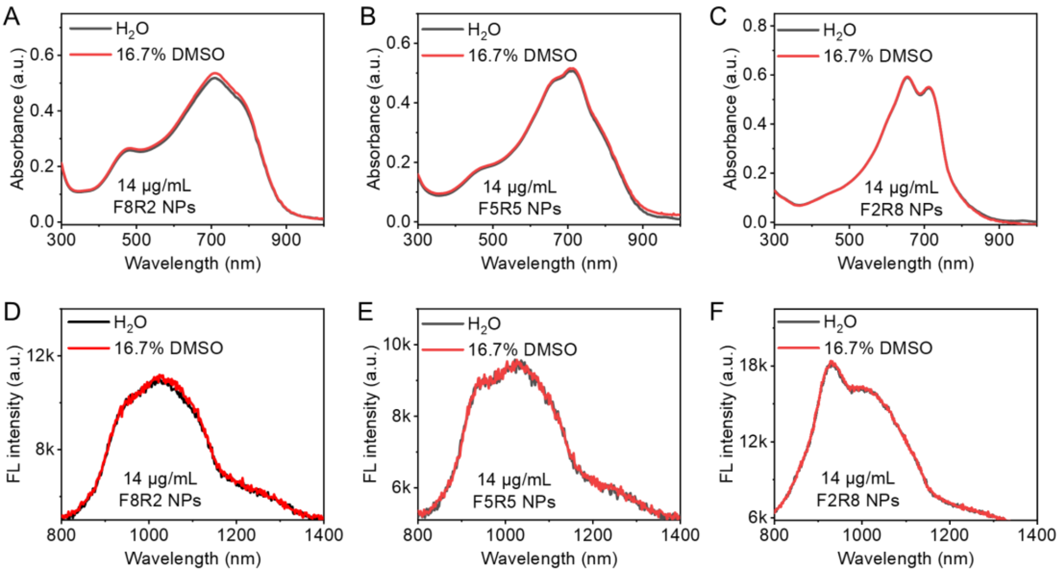

Figure S25. Optical properties of aqueous nanoparticle dispersions (14 μg/mL) after adding DMSO. Absorbance spectra changes of F8R2 NPs (A), F5R5 NPs (B), and F2R8 NPs (C) after adding 16.7% DMSO. Fluorescence spectra changes of F8R2 NPs (D), F5R5 NPs (E), and F2R8 NPs (F) after adding 16.7% DMSO.

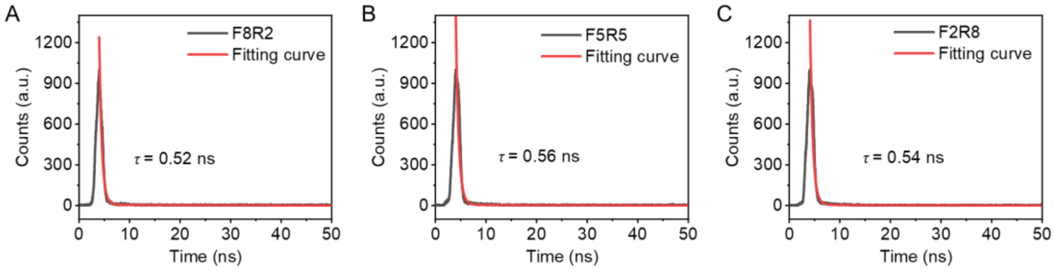

Figure S26. PL lifetimes of F8R2 (A), F5R5 (B), and F2R8 (C) aggregates (670 nm EPL laser excitation).

Table S6. Summary of radiative and nonradiative decay rates estimated from the PLQY and fluorescence lifetime.

Sample	PLQY(Φ)	τ (ns)	k_r (10^7 s^{-1})	k_{nr} (10^9 s^{-1})
F8R2 NPs	0.0044	0.52	0.85	1.91
F5R5 NPs	0.0065	0.56	1.16	1.77
F2R8 NPs	0.0168	0.54	3.11	1.82

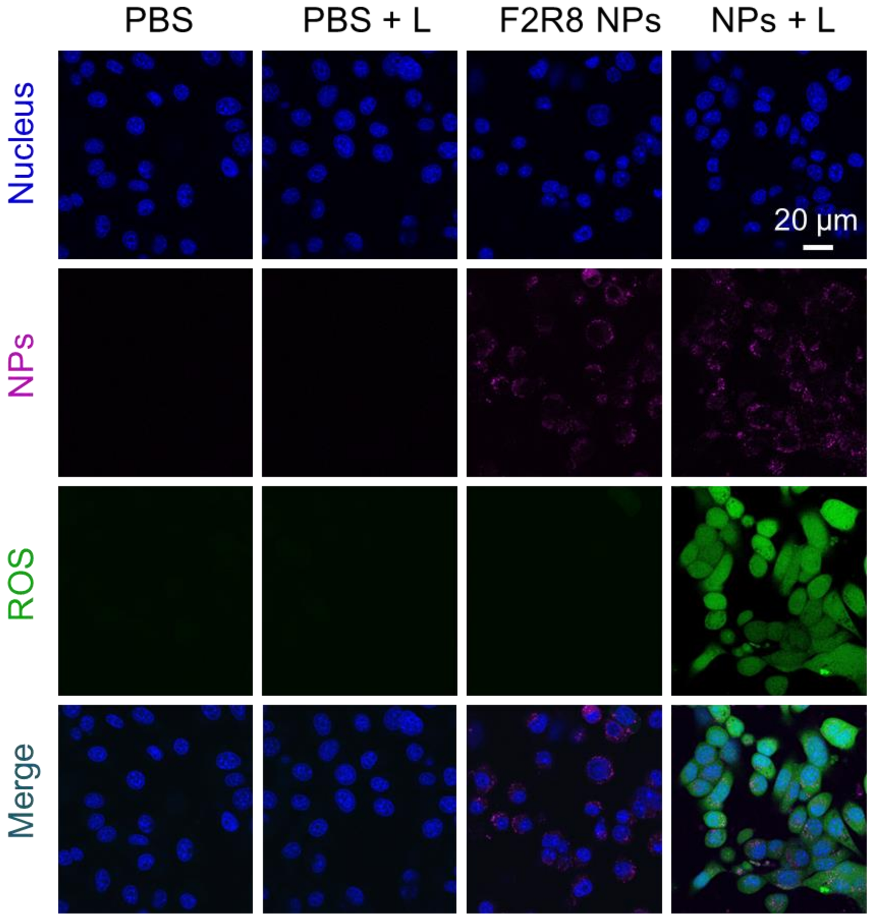

Figure S27. Confocal fluorescence images of intracellular ROS generation in 4T1 cells treated with Cy3-labeled F2R8 NPs under different conditions. DCFH-DA was used as the ROS probe, and Hoechst 33342 was used for nuclear staining. The NPs were prepared using F127 and DSPE-PEG-Cy3 at a mass ratio of 9:1. The fluorescence signal of the NPs originates from the incorporated Cy3 fluorophores.

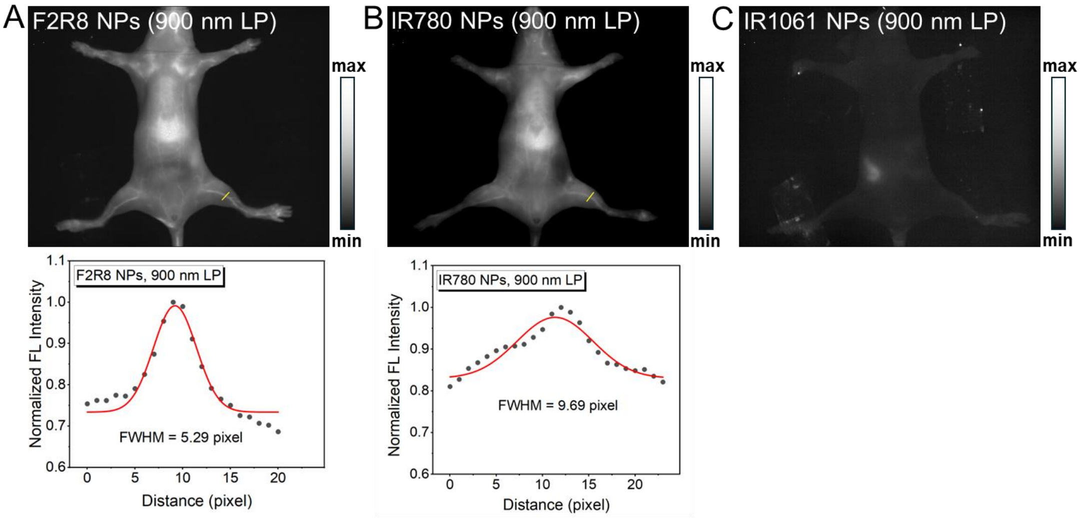

Figure S28. Whole-body vascular imaging of mice following injection of an equal dosage of F2R8 NPs (A), IR780 NPs (B), and IR1061 NPs (C), acquired using a 900

nm LP filter, along with the corresponding FWHM analysis.

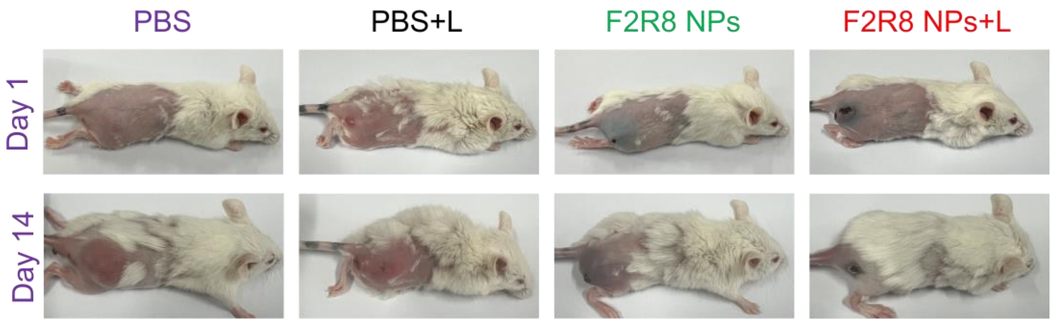

Figure S29. The images of mice being received different treatments on day 1 and day 14.

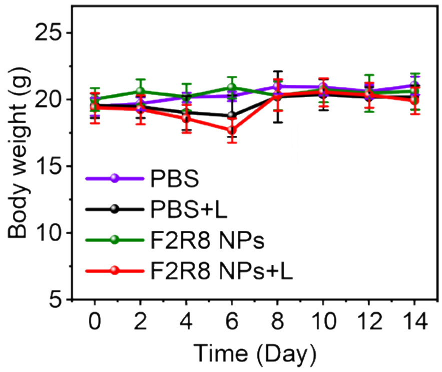

Figure S30. Body weight of the mice in different treatment groups (*n*=5).

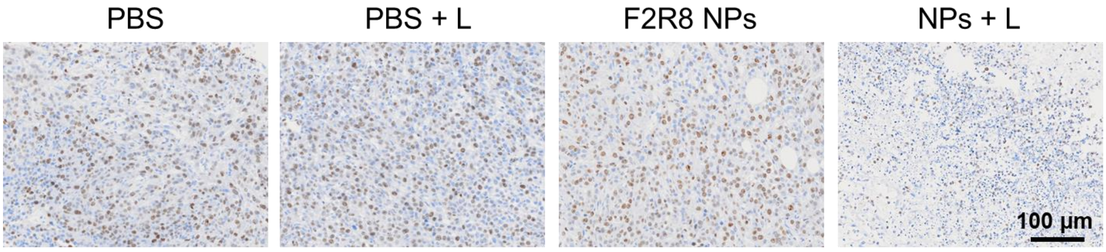

Figure S31. Ki67 immunohistochemical staining of tumor sections from different groups at 24 h post-treatment.

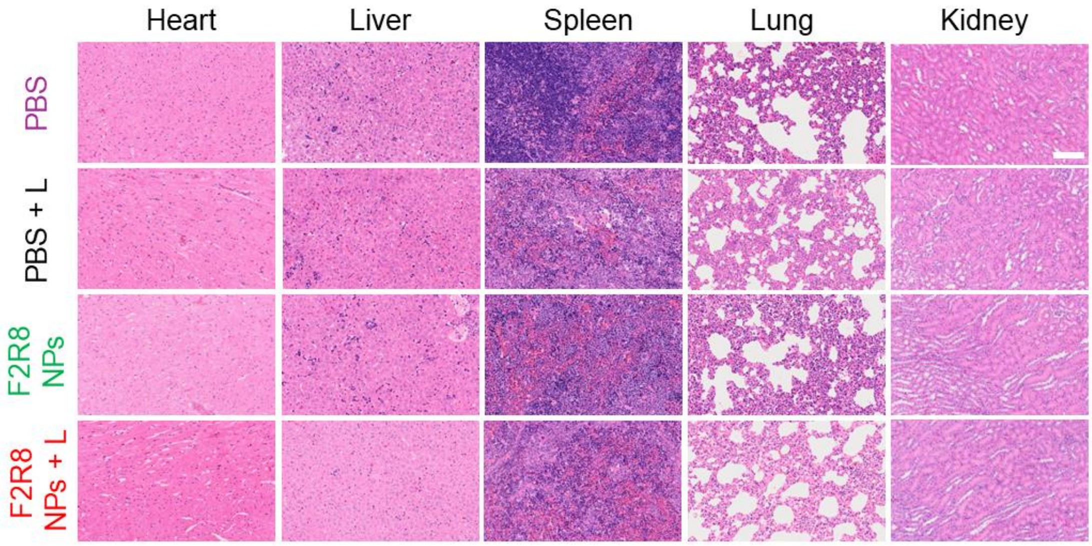

Figure S32. H&E staining of major organs excised from different treatment groups. Scale bar = 100 μm.

10. Reference

(1) Frisch, M.; Trucks, G.; Schlegel, H. B.; Scuseria, G.; Robb, M.; Cheeseman, J.; Scalmani, G.; Barone, V.; Petersson, G.; Nakatsuji, H. Gaussian 16, Gaussian. *Inc., Wallingford CT* **2016**, *2016*.

(2) Humphrey, W.; Dalke, A.; Schulten, K. VMD: visual molecular dynamics. *J. Mol. Graph.* **1996**, *14* (1), 33-38.

(3) Emamian, S.; Lu, T.; Kruse, H.; Emamian, H. Exploring nature and predicting strength of hydrogen bonds: a correlation analysis between atoms - in - molecules descriptors, binding energies, and energy components of symmetry - adapted perturbation theory. *J. Comput. Chem.* **2019**, *40* (32), 2868-2881.

(4) Lee, K. W.; Gao, Y.; Wei, W. C.; Tan, J. H.; Wan, Y.; Feng, Z.; Zhang, Y.; Liu, Y.; Zheng, X.; Cao, C. Anti - quenching NIR - II J - aggregates of benzo [c] thiophene fluorophore for highly efficient bioimaging and phototheranostics. *Adv. Mater.* **2023**, *35* (20), 2211632.
